# Extracellular Vesicles in Colorectal Cancer: From Tumor Growth and Metastasis to Biomarkers and Nanomedications

**DOI:** 10.3390/cancers15041107

**Published:** 2023-02-09

**Authors:** Larissa Kotelevets, Eric Chastre

**Affiliations:** Sorbonne Université, INSERM, UMR_S938, Centre de Recherche Saint-Antoine (CRSA), 75012 Paris, France

**Keywords:** extracellular vesicles, exosomes, ectosomes, microvesicles, colorectal cancer, metastasis, miRNA, lncRNA, diagnosis, nanomedicine

## Abstract

Colorectal cancer (CRC) is a leading public health concern due to its incidence and high mortality rates, highlighting the requirement of an early diagnosis. Evaluation of circulating extracellular vesicles (EVs) might constitute a noninvasive and reliable approach for CRC detection and for patient follow-up because EVs display the molecular features of the cells they originate. EVs are released by almost all cell types and are mainly categorized as exosomes originating from exocytosis of intraluminal vesicles from multivesicular bodies, ectosomes resulting from outward budding of the plasma membrane and apoptotic bodies’ ensuing cell shrinkage. These vesicles play a critical role in intercellular communications during physiological and pathological processes. They facilitate CRC progression and premetastatic niche formation, and they enable transfer of chemotherapy resistance to sensitive cells through the local or remote delivery of their lipid, nucleic acid and protein content. On another note, their stability in the bloodstream, their permeation in tissues and their sheltering of packaged material make engineered EVs suitable vectors for efficient delivery of tracers and therapeutic agents for tumor imaging or treatment. Here, we focus on the physiopathological role of EVs in CRCs, their value in the diagnosis and prognosis and ongoing investigations into therapeutic approaches.

## Simple Summary

Almost all cell types produce extracellular vesicles that, according to their size, subcellular origin and release pathways, are mainly categorized as exosomes, ectosomes and apoptotic bodies. These vesicles exert a critical role in intercellular communication during physiological and pathological processes through the delivery of their cargo. Extracellular vesicles display the molecular features of the cells they originate and thus, they might serve as a basis for the noninvasive diagnosis of cancer or for patient follow-up using liquid biopsies. Furthermore, extracellular vesicles can be engineered for the selective and efficient delivery of molecular tracers and therapeutic agents for tumor imaging or treatment. This review provides an overview of the role of extracellular vesicles in the progression of colorectal cancers, in remodeling target tissue to facilitate premetastatic niche formation, their predictive value for the diagnosis and prognosis of colorectal cancer and the ongoing evaluations of their potential use as nanomedications.

## 1. Introduction

**Figure 1:**
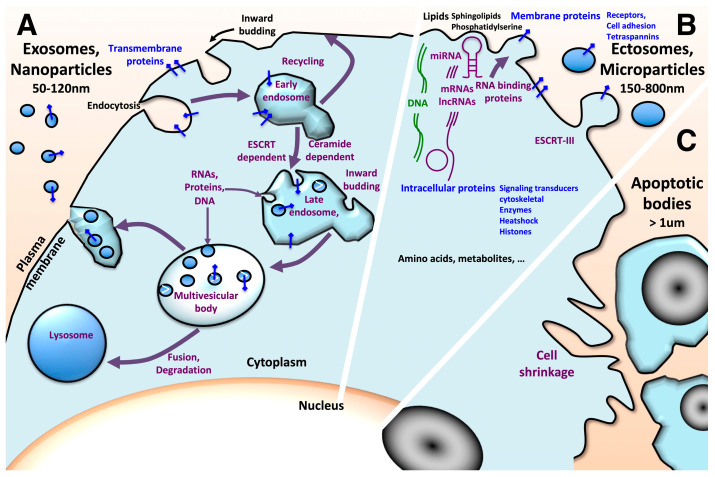
Schematic overview of biogenesis of the three main types of membrane extracellular vesicles. (**A**) Exosomes originate from the inward membrane budding of late endosomes leading to intraluminal vesicle accumulation and the formation of multivesicular bodies. This process involves machineries that segregate cargoes into microdomains of the membranes of multivesicular bodies. This can be achieved via the ESCRT pathway. The ESCRT-0 complex interacts and clusters ubiquitinated transmembrane proteins on microdomains and it interacts with the ESCRT-I complex, which recruits ESCRT-II. ESCTR-I/ESCTR-II initiate local budding of the vesicular membrane, triggering and recruiting ESCRT-III with accessory proteins that promote scission of membrane vesicles with sequestered cytosol. Alternative pathways independent of the ESCRT complexes were evidenced. This includes the syndecan-syntenin-ALIX pathway, which still requires ESCRT-III for membrane fission, and the ceramide pathway. The neutral type II sphingomyelinase hydrolyses sphingomyelin to ceramide, leading to an accumulation of ceramide that triggers curvature of the endosomal membrane [7]. Tetraspanins, a family of transmembrane proteins, organize membrane microdomains and contribute to cargo-sorting. (**B**) Ectosome biogenesis also involves membrane proteins sorting through tetraspanins; their clustering in subdomains promotes outward budding of the plasma membrane. The recruitment of TSG101 (subunit of ESCTR-I complex) mobilizes the ESCRT-III complex and induces the release of the vesicles. The cargoes of the exosomes and ectosomes are plasma membrane proteins, including receptors (e.g., epidermal growth factor receptor EGFR and hepatocyte growth factor receptor c-MET), cell adhesion molecules (e.g., integrins and cadherins), tetraspanins (e.g., CD9 and CD81), cytoplasmic proteins, including signaling transducers (e.g., β-catenins, GTPase KRas and proto-oncogene tyrosine-protein kinase Src), cytoskeletal proteins (e.g., actin and tubulin), chaperones (e.g., heat shock proteins HSP70 and HSP90) and metabolic enzymes, but also nucleic acids including DNA and RNA (mRNAs and ncRNAs). RNA binding proteins exert a critical role in the selective sorting and the depletion/enrichment of RNA in extracellular vesicles. Besides EV diversity related to sorting machineries, their cellular ultrastructures and polarization, e.g., apical vs. basolateral poles, might contribute to the cargo content as well as to the bioavailability of the released EVs and their biological impact. (**C**) Apoptotic bodies result from cell shrinkage. They contain lipids, proteins, nucleic acids and even micronuclei and organelles.

Extracellular vesicles (EVs) are gaining greater interest as they prove to orchestrate intercellular communication and exchanges through the transfer of lipids, nucleic acids, proteins and metabolites under pathophysiological conditions. EVs secretion is an evolutionarily conserved process that occurs in lifeforms from bacteria and archaea to protists and multicellular eukaryotic organisms, further highlighting their critical importance in information transfer. In mammals, EVs are found in all biological fluids, including blood, urine, saliva, cerebrospinal fluid, amniotic fluid, breast milk and seminal fluid. Based on their size, subcellular origins, release pathways and cargo content, extracellular vesicles are mainly categorized as exosomes, ectosomes and apoptotic bodies. Some guidelines concerning the identification and characterization of extracellular vesicles are regularly updated [1,2,3]. Exosomes, also known as nanovesicles, are characterized by a diameter ranging 50–120 nm. They originate as intraluminal vesicles from the inward budding of endosomal membrane from endocytic vesicles, leading to the formation of multivesicular bodies. Multivesicular bodies (MVBs) constitute a step in the degradative lysosome pathway. The alternative route concerns the release of intraluminal vesicles via exocytosis upon fusion of multivesicular bodies with the plasma membrane (Figure 1) [4,5]. This process involves pathways dependent on and independent of ESCRT (endosomal sorting complexes required for transport) machinery [6,7,8]. ECSRT-0, -I and –II allow the sorting of ubiquitinated proteins. ESCRT-III coils as spiral oligomers around the site of membrane constriction prior to membrane cleavage.

Ectosomes, also known as microvesicles or microparticles, are 150–800 nm vesicles. They result from outward budding of the plasma membrane. This process involves the ESCRT-III complex for membrane fission. Apoptotic bodies are larger vesicles with a size of 500 nm–2 μm. They originate from apoptotic cell disassembly. They may contain organelles, micronuclei and DNA fragments. These large vesicles are engulfed by macrophages, parenchymal cells and tumor cells, and they are degraded within phagolysosomes. Nevertheless, the biological impacts of apoptotic bodies are poorly documented [9].

Further subsets of nanoparticles characterized by distinct size, cargo and tissue uptake were recently described. Exo-L, or large exosome vesicles (90–120 nm), may represent noncanonical exosomes; Exo-S, or small exosome vesicles (60–80 nm) are likely canonical exosomes [10]. Exomeres (35 nm) constitute an abundant population of nonmembranous nanoparticles that are enriched in proteins involved in metabolism (glycolysis and mTORC1 metabolic pathways) [11,12]. Supermeres (a supernatant of exomeres) are smaller nonmembranous entities (25–35 nm) characterized by distinct cargo (glycolytic enzymes, miR-1246, TGFβ-induced protein TGFBI, hepatocyte growth factor receptor MET, glypican 1 and Argonaute RISC catalytic component 2 AGO2) and a greater uptake in vivo compared to small extracellular vesicles and exomeres [13].

Extracellular vesicles are rich in a set of lipids and proteins including annexins, tetraspanins and heat-shock proteins, but they also carry different sets of nucleic acids including DNA, mRNAs, noncoding RNAs (ncRNAs), miRNAs and long noncoding RNAs (lncRNAs) that are selectively sorted. These cargoes are transferred to target cells via fusion with the plasma membrane or through endocytosis [6]. Nevertheless, the MISEV2018 (consortium “minimal information for studies of extracellular vesicles”) release concludes that due to the different cellular sources in use to investigate EVs and the different isolation approaches, it was not possible to propose specific and universal EV subtypes.

Extracellular vesicles proved to be involved in many human diseases, including neurodegenerative disorders, diabetes and heart disease. In cancer, EVs can act not only as paracrine factors to drive tumor microenvironment changes favoring tumor growth, invasiveness, angiogenesis and immunomodulation, but also as systemic mediators to prepare premetastatic niches. Besides microenvironment remodeling during carcinogenesis, another side effect of extracellular vesicles is venous thromboembolism, a frequent complication that markedly increases the risk of mortality and degrades the quality of life for patients [14]. As a matter of fact, the exposure of microparticles bearing tissue factor (coagulation factor III) derived from tumors to factor VII circulating in blood might initiate the coagulation cascade, leading to thromboembolism. Furthermore, platelet–colorectal cancer cell interactions potentiate the release of platelet-derived procoagulant EVs [15].

On another note, EVs carry the molecular signature from cells they originate from, and thus, they might serve as a basis for diagnostic or follow-up purposes. Furthermore, the biocompatibility and stability of EVs in the bloodstream, their permeation in tissues, the sheltering and cloaking of packaged material and their uptake by cancer cells make engineered EVs suitable vectors for the selective and efficient delivery of molecular tracers and therapeutic agents for tumor imaging or treatment.

The present review focuses on the physiopathological role of EVs in colorectal cancers (CRCs), their value in the diagnosis and follow-up of patients with CRC, and ongoing investigations in their beneficial use in therapeutic approaches. Accordingly, colorectal cancer is a major cause of cancer morbidity and mortality in Western countries. It is the 3rd most frequent cancer diagnosed in both women and men in the United States, and in Europe, it is the 2nd and the 3rd most frequent cancer in women and men, respectively. It has been estimated that 150,000 and 520,000 new cases are diagnosed annually in the United States and in Europe, respectively, where this cancer is responsible for approximately 53,000 and 250,000 related deaths, respectively [16,17,18]. Liver metastases represent the main cause of colorectal cancer-related mortality. When colorectal cancer is localized, the five-year survival rate is about 90%, but it falls to nearly 14% for patients with metastatic disease [16], highlighting the requirement of an early diagnosis. For this purpose, screening programs were developed and guidelines were created according to individual risk [19]. For high-risk individuals, a colonoscopy is recommended. In absence of evident risk factor, for healthy individuals between 50 and 74 years of age, disease screening is based on a fecal occult blood test with a two-year periodicity, which, if positive, is complemented by colonoscopy. Although the selectivity and the specificity of such tests are also found in immunochemical testing (fecal immunochemical testing FIT), false positives leading to unnecessary colonoscopies with potential risks of complications remain. Furthermore, although noninvasive, FIT requires population adherence and a lack of reluctance toward this procedure.

To increase individual compliance with colorectal cancer screening programs, alternative noninvasive approaches should be developed. Among them, the evaluation of circulating extracellular vesicles is to be considered. Especially in terms of the underlying molecular defects, CRC is one of the best characterized. Colorectal cancers evolve through the stepwise accumulation of genetic alterations leading from normal epithelia to aberrant crypt foci, adenoma, carcinoma and metastatic disease [20,21], and they follow three molecular pathways, characterized by (i) chromosomal instability (CIN), (ii) high microsatellite instability (MSI-H) or (iii) CpG island methylator phenotype (CIMP), that can lead to the MSI phenotype. These pathways involve different sets of gene dysregulations related to similar signaling pathways, including Wnt, KRAS, SMAD mutations for CIN tumors, β-catenins, PIK3CA/PTEN, and TGFβ-R2 for MSI tumors. Chronic diseases, including intestinal inflammation, are also associated with an increased risk of colon cancer [21,22]. It should be noted that although the genetic defects involved in colitis-associated cancers are similar to those of sporadic CRCs, the sequence of events differs, e.g., P53 inactivation occurs early, whereas APC mutation is a late event [22]. A more detailed classification of primary colorectal cancers taking into account intrinsic gene expression profiles and resulting in the four biologically distinct consensus molecular subtypes (CMS1–4) was recently established to facilitate the translation of molecular subtypes into the clinic [23]. These signatures might serve as the basis for the selective screening, follow-up and/or treatment of patients with colorectal cancer.

## 2. Role of Extracellular Vesicles in Colorectal Tumor Progression

### 2.1. CRC-Derived EVs in Microenvironment Remodeling

The crosstalk between colonic cancer cells, colonic epithelial cells, fibroblasts, endothelial cells and cells of the immune system are critical in remodeling colonic mucosa and settling microenvironments favoring tumor growth [24,25].

The use of in vitro models has provided major insights into the leading role of EVs in these interactions, their underlying mechanisms and their biological significance. In this sense, frizzled-10 in exosomes from the human colon cancer Caco-2 and SW620 cells is able to reprogram and confer the epithelial-mesenchymal transition (EMT) phenotype to the normal colonic epithelial HCEC-1CT cell line (Table 1) [26]. Similarly, miR-224-5p from colorectal cancer SW620 cell-derived exosomes triggers a malignant phenotype characterized by enhanced viability, proliferation, migration and invasiveness to the nontumorigenic CCD 841 CoN cell line through the downmodulation of the chemokine-like factor CMTM4 [27]. This cell line was established from healthy human colonic tissue, but according to morphological features and the absence of keratin, its epithelial origin is lacking. Tetraspanin 6 (Tspan6), frequently downregulated in CRC, proved to suppress early stages of intestinal tumor development in APC^Min/+^ mice [28] (Table 1). Mechanistically, Tspan6 forms a tripartite complex involving the scaffolding molecule syntenin and the transmembrane form of TGF-α (tmTGF-α). These interactions impair the recruitment of tmTGF-α into multivesicular bodies and its subsequent release as extracellular vesicles into the extracellular space, leading to stimulation of the EGFR pathway [28]. Interestingly, Tspan6 expression in tumors is a predictive marker of response to the EGFR inhibitor cetuximab in CRC patients.

The oncogenic status and the phenotype of CRC cells also affect EVs’ cargo and their biological significance. The use of the human colon cancer DLD-1 cell line bearing a heterozygous mutation of KRAS, as well as isogenic derivatives with wild-type or homozygous KRAS mutation, revealed the enrichment of this oncoprotein in exosomes concurrently with other tumor-promoting proteins, including EGFR and SRC family kinases [29]. Interestingly, these exosomes induce anchorage-independent growth of DLD-1 cells with wild-type KRAS. GTPase KRas activation proved to also affect miRNA sorting. MiR-10b levels are selectively increased in wild-type KRas exosomes, whereas miR-100 accumulates in mutant KRas exosomes [30]. The sorting of this latter miRNA in exosomes involves neutral sphingomyelinase that produces ceramide.

Exosomes generated by early- and late-stage CRC cells differently affect the functional reprograming of quiescent fibroblasts. In contrast with EVs released by mesenchymal-like CRC cell lines, EVs derived from epithelial CRC cell lines suppress the TGFβ-driven fibroblast differentiation into myofibroblast. The latter EVs are enriched in miR-200, which depletes the transcription repressor ZEB1 in fibroblasts [31]. These observations might account for the accumulation of myofibroblastic stroma in the mesenchymal CMS4 CRC subset characterized by TGFβ pathway activation. In the same way, the exosomes derived from the human colon cancer SW480 cell line promote a pro-proliferative (increased expression of protein S100-A6 and farnesyl-diphosphate synthase) and pro-angiogenic (interleukin-8 IL-8, Ras-related GTP-binding protein RAB10 and N-Myc downstream regulated 1 NDRG1) phenotype to activated fibroblasts, whereas exosomes derived from the isogenic SW-620 metastatic counterpart drive a pro-invasive phenotype characterized by an increased accumulation of PDLIM1 (PDZ and LIM domain protein 1), MYO1B (unconventional myosin-Ib), MMP11 (stromelysin-3), basigin and ADAM10 (disintegrin and metalloproteinase domain-containing protein 10) [32].

### 2.2. CRC-Derived EVs in Angiogenesis

The activation of endothelial cells and the angiogenic switch constitutes an essential step, once tumors have reached a size of about 1 mm, to provide tumor cells with nutrients and oxygen and to remove metabolic wastes. Several angiogenic factors secreted by cancer cells, including the vascular endothelial growth factor family of peptides (VEGFs) promote this process. Hypoxia is also a potent inducer of EVs produced by cancer cells, and a series of miRNAs conveyed in tumor exosomes are involved in angiogenesis. This includes the miR-221-3p released by the human colon cancer HCT-116 cells that triggers the proliferation, migration and tubulogenesis of endothelial cells through the depletion of SOCS3 (suppressor of cytokine signaling 3) transcripts and the subsequent upregulation of VEGFR [33] (Table 2). For its part, MiR-21-5p targets KRIT1 (Krev interaction trapped protein 1) in endothelial cells, resulting in the activation of the β-catenin signaling pathway as well as the upregulation of VEGFa and Ccnd1 (cyclin D1); thus, it promotes angiogenesis and vascular permeability [34]. Interestingly, this oncomiR is induced by hypoxia [35]. 

**Table 1:** Partial list of proteins identified in exosomes with evidence of biological effects and clinical implications in colorectal cancer.

Protein	Full Name	Function	Producing Cells	Exosome Isolation	Recipient Cells	Biological Effect	Clinical Implication	References
ANGPTL1	Angiopoietin-like 1	Member of vascular endothelial growth factor family	Human CRC, SW5620, cells overexpressing ANGPTL1	Conditioned medium, colonic tissue; differential centrifugation	Kupffer cells	Kupffer cells reprogramming, decreased MMP9 release	Attenuates CRC liver metastasis and impedes vascular leakiness	[36]
DNAJB8	DnaJ heat shock protein family (Hsp40) member B8	Chaperone	Blood samples from patients with CRC; SW480 and SW620 cell derivatives resistant to oxaliplatin after long term treatment	Blood samples, conditioned medium from SW480 and SW620 cell lines; density gradient centrifugation	Parental SW480 and SW620 cell lines	Inhibits P53 ubiquitination and degradation leading to MDR1 upregulation and resistance to oxaliplatin	Transfer of resistance to oxaliplatin	[37]
FZD10	Frizzled-10	Member of the Wnt receptor family	Human colon cancer cells (Caco-2)	Conditioned medium; Total Exosome Isolation kit (Invitrogen)	Colonic epithelial cells	Activation of Wnt/β-catenin signaling pathway; EMT	Invasiveness	[26]
HSPC111, NOP16	Nucleolar protein 16	Nucleolar protein	Human colon cancer cell lines	Conditioned medium; ExoQuick-TC Exosome Isolation kit (System Biosciences)	Hepatic stellate cells	Education of stellate cells into cancer associated fibroblasts (CAFs)	Liver metastasis	[38]
HUR, ELAV1	Hu antigen R, ELAV-like protein 1	RNA binding protein	Human colon cancer HCT116 cell line	Conditioned medium, colonic tissue; differential centrifugation	Human bronchial epithelial cell line BEAS-2B	Stabilizes c-Myc transcripts and downregulates p21 expression	Increased proliferation and migration of bronchial cells. Tissue remodeling? Premetastatic niche formation?	[39]
IDH1	Isocitrate dehydrogenase 1	Glucose metabolism	Human colorectal cancer HCT8 cell derivatives resistant to 5- fluorouracil (5-FU)	Conditioned medium; ultracentrifugation	Parental HCT8 cell line	Glycometabolism reprogramming; increased intracellular levels of NADPH	Transfer of resistance to 5-FU	[40]
IRF2	Interferon regulatory factor 2	Transcription factor; inhibits IRF1-mediated transcriptional activation	Blood samples from patients with CRC; mouse colon cancer CT26 cell lines	Density gradient centrifugation	Macrophages	VEGF-C release	Lymphangiogenesis and lymph node metastasis	[41]
ITGBL1	Integrin beta-like 1	Beta integrin-related protein	Human colon cancer cell lines	Conditioned medium; differential centrifugation	Hepatic fibroblasts and stellate cells	Interacts with TNFAIP3, leading to NF-κB signaling pathway activation	Premetastatic niche formation	[42]
KRAS (activated)	GTPase KRas	Member of the small GTPase superfamily; proto-oncogene, upstream regulator of the RAS/MAPK and PI3K/Akt pathways	Human colon cancer cells (DLD1 cells and isogenic derivatives)	Conditioned medium; filtration, differential centrifugation	Human colon cancer cells	Mutant KRas is preferentially enriched in EVs, concurrently with EGFR, RAP1, SRC, LYN, integrins, cortactin, and p120 catenin; promotes anchorage independent growth of colon cancer cells with wild type KRAS	Tissue remodeling? Tumor niche development? Remote impact on metastatic sites?	[29]
GAS6	Growth arrest specific protein 6	Ligand of AXL receptor tyrosine kinase	Tumor perivascular cells	Conditioned medium, colonic tissue; ExoQuick-TC Exosome kit (EXOTC50A-1, System Biosciences)	Endothelial progenitor cells	Recruits endothelial progenitor cells via activating the Axl pathway	Tumor revascularization after antiangiogenic therapy withdrawal	[43]
p-AKT	Phosphorylated AKT	Ser/Thr kinase	Human colorectal cancer HCT116 and LoVo cell lines	Conditioned medium; Differential centrifugation	Hepatic stellate cells	Stimulates interleukin-6 (IL-6) release by stellate cells leading to enhanced lactate metabolism of hypoxic CRC cells	Resistance to SN38 (active metabolite of irinotecan)	[44]
p-ERK	Phosphorylated extracellular signal-regulated kinase; mitogen-activated protein kinase	Ser/Thr kinase; member of the MAP kinase family	Human colorectal cancer HCT116 and LoVo cell lines	Conditioned medium; Differential centrifugation	Hepatic stellate cells	Stimulates IL-6 release by stellate cells leading to enhanced lactate metabolism of hypoxic CRC cells	Resistance to SN38 (active metabolite of irinotecan)	[44]
p-Stat3	Phosphorylated signal transducer and activator of transcription 3	Transcription activator	Human colorectal cancer RKO cell derivatives resistant to 5-FU after long-term treatment	Conditioned medium, colonic tissue; differential centrifugation	Parental RKO and HCT116 colon cancer cells	Decreased apoptosis	Transfer of resistance to 5-FU	[45]
tmTGF-α	Pro-transforming growth factor alpha	Ligand of EGFR, transmembrane form of TGF-α	Intestinal organoids from APC^Min/+^ mice	Conditioned medium; differential centrifugation	Mouse intestinal organoids	Activation of EGFR pathway; autocrine growth regulation; tetraspanin 6 impairs the recruitment of tmTGF-α into EVs	Resistance to anti-EGFR inhibitor (cetuximab)?	[28]
Wnt3a	Protein Wnt-3a	Member of the Wnt family; ligand of frizzled receptor	CAFs isolated from human CRC	Conditioned medium; Total Exosome Isolation Kit (Invitrogen)	Human colon cancer HT-29 and SW620 cell lines	Activates β-catenin signaling pathway; triggers colon cancer cell dedifferentiation and stemness	Resistance to oxaliplatin and 5-FU	[46]

Exosomal miR-1229 derived from HCT-116 colon cancer cells or from blood samples of patients with CRC triggers angiogenesis by targeting the Ser/Thr kinase HIPK2 (Homeodomain Interacting Protein Kinase 2), which acts as either a corepressor or a coactivator of transcription factors. Downregulation of HIPK2 in human umbilical vein endothelial cells (HUVECs) enhances MEF2C transcriptional activity and VEGF accumulation. High levels of this miRNA in exosomes are associated with poor overall survival in CRC patients [47]. MiR-1246 accumulation is decreased within colorectal tumor tissues and cell lines, but it as well as TGFβ are enriched in circulating EVs. MiR-1246 and TGFβ act together to stimulate endothelial cell proliferation, migration and tubulogenesis. MiR-1246 depletes PML (promyelocytic leukemia protein), impairing Smad2/3-induced endothelial cell quiescence and favoring the Smad1/5/8 pathway, which is further reinforced by TGFβ [48]. Extracellular vesicles released by tumor perivascular cells are also involved in angiogenesis through the release of exosomes containing Gas6, the ligand for tyrosine-protein kinase receptors AXL [43,49].

### 2.3. Impact of CRC-Derived EVs on Immune Response

Regarding crosstalk with immune cells and immune escape, tumor-derived EVs were proven to reprogram and/or affect the activities of cells involved in innate and adaptative immunity [50,51]. For instance, miR-424 EVs suppress the CD28-CD80/86 costimulatory pathway in tumor-infiltrating T cells and dendritic cells, resulting in resistance to immune checkpoint blockades [52]. The cytotoxic activity of natural killer cells is inhibited by EVs containing the lncRNA SNHG10 released by an epithelial–mesenchymal transition (EMT) model of SW480 cells [53]. Tumor EVs also promote an immunosuppressive microenvironment by triggering macrophage polarization to M2-like phenotypes with PD-L1 expression. Accordingly, whereas the classically activated M1 macrophages exhibit cytotoxic activities against cancer cells, the M2 alternative polarization is involved in the elimination of pathogens, angiogenesis and tissue remodeling and repair. These tumor-associated macrophages (TAMs) are known to impair the inflammatory response and to favor tumor growth [51,54]. In this concern, the enhanced abundance in PD-L1^+^CD206^+^ macrophages leads to decreased T cell activity in CRCs [55]. Mechanistically, CRC-derived EVs increase PD-L1 expression in tumor-associated macrophages (TAMs) in at least two ways. On one hand, miR-21-5p and miR-200a exhaust the transcripts of the PTEN tumor suppressor, leading to activation of the AKT signaling pathway, and on the other hand, miR-21-5p targets SOCS1, which negatively controls the STAT1 signaling pathway. Furthermore, M2 macrophage-derived EVs contribute to CRC immune escape through miR-155-5p transfer to colon cancer cells. This miRNA downmodulates ZC3H12B (zinc finger CCCH-type containing 12), which is thought to function as an RNAse, leading to upregulation of IL-6 in CRC cells and inhibition of T cell immune response [56]. Interestingly, this miRNA released by M2 macrophages promotes CRC cell migration and invasion by targeting the tumor suppressor BRG1, which regulates gene transcription via chromatin remodeling [57]. M2 macrophage-derived EVs are also enriched in miR-186-5p, which depletes the Rho GTPase/tumor suppressor DLC1 (deleted in liver cancer 1 protein), leading to activation of β-catenin signaling, enhanced CRC cell proliferation and induction of EMT [58]. Surprisingly, TAM-EVs derived from MC38 CRC mouse models display a proteomic and lipidomic signature that was associated with inflammation and immune response through Th1/M1 macrophage polarization [59]. Besides tumor growth and invasiveness, M2 macrophages facilitate remote CRC cell implantation and the metastatic process (Table 2 and Section 3.2). Colon cancer cell-derived exosomes also exert immunosuppressive activity by promoting expansion of the regulatory T cell (T-reg CD4^+^CD25^high^Foxp3^+^) population through miR-208b’s targeting of PDCD4 (programmed cell death factor 4) in CD4^+^ T cells [60].

Likewise, modeling of the tumor microenvironment involves remote activity of EVs on protumoral immune cells. Exosomes derived from the mouse colon cancer CT-26 stem cells xenografted in syngeneic Balb/c mice reach the bone marrow, where the exosomal 5-triphosphate RNA cargo triggers pattern recognition response with bone marrow-derived neutrophils, and the release of IL-1β sustains their survival. These primed neutrophils are then recruited to the tumor site by the CXCL1 and CXCL2 cytokines secreted from cancer cells, and they enhance tumorigenesis via IL-1β [61].

Extracellular vesicles produced by platelets exert a dual role in CRC progression [62]. Platelets interact with cancer cells through cadherin-6, leading to the release of EVs expressing platelet markers, tumor markers, or both. On one hand, these microparticles recruit monocytes producing IFN-γ and IL-4, which are involved in the tumoricidal function of macrophages, via the chemoattractants RANTES/CCL5, MIF (macrophage migration inhibitory factor), CCL2 and CXCL12; thus, they suppress primary tumor growth. On the other hand, circulating microparticles activate endothelial cells and platelets, facilitate the interaction of cancer cells with the endothelium and induce EMT; thus, they promote metastasis [62].

### 2.4. Microbiota-Derived EVs in Colorectal Carcinogenesis

The extracellular vesicles generated by the microbiota also contribute to the control of colorectal carcinogenesis through the modulation of tissue integrity and immune response [63]. Accordingly, chronic intestinal inflammation is a significant risk factor for colon cancer development. For example, EVs derived from *Akkermansia muciniphila*, a gut commensal bacterium curtailing dextran sulfate sodium (DSS), induced colitis in mice [64]. This effect seems to be related to the maintenance of intestinal barrier integrity and decreased inflammation [65]. The outer membrane vesicles from *Bacteroides fragilis* produce a capsular polysaccharide, which induces regulatory T cells and mucosal tolerance that alleviates colitis in experimental models [66]. *Clostridium butyricum*-derived EVs improve the remission of murine colitis and polarization of macrophages to the M2 phenotype [67]. Similar observations were made with the lactic acid commensal bacterium *Pediococcus pentosaceus* [68]. In contrast, *Fusobacterium nucleatum*-derived extracellular vesicles promote the migration of the human colon cancer Caco-2 cells in vitro [69]. This oral anaerobic opportunistic pathogen is enriched in colon tumors, interacting with E-cadherins and Gal/GalNAc on cancer cell surfaces. More complex features were observed with the extracellular vesicles released from the human commensal gut bacteria *Bacteroides thetaiotaomicron* that affect not only host immune pathways in a cell type specific manner but also according to pathophysiological status (healthy individuals vs. patients with ulcerative colitis) [70].

### 2.5. Depletion of Tumor-Suppressive ncRNAs in CRC Cells through Exosomes

Besides their role in intercellular communication, EVs might also favor tumor growth by selectively sorting and exhausting tumor-suppressive cargo. MiR-193a is downregulated in colorectal tumors, but it accumulates in circulating exosomes of patients with colorectal cancer in a stage-dependent manner [71]. The major vault protein (MVP), a component of the multi-subunit ribonucleoprotein complex Vault, is required for the packaging of miR-193 into exosomes and for its reduced cytoplasmic accumulation. Downregulation of MVP is associated with an increased intracellular level of miR-193a that triggers cell cycle G1 arrest and impairs the growth of the human colon cancer SW620 in nude mice by targeting caprin-1 (cell cycle associated protein 1), an RNA binding protein that upregulates Ccnd2 and c-Myc [71]. Similarly, miR-8073 is present in exosomes and predominantly exported from human colon cancer HCT-116 cells compared to the control human colonic epithelial HCOEpiC cell line. An miR-8073 mimic selectively decreases the proliferation of various types of cancer cells, but it does not affect normal cells. This tumor-suppressive activity might be related to the targeting of forkhead box protein M1 (FOXM1), methyl-CpG-binding domain protein 3 (MBD3), cyclin D1, kallikrein-10 (KLK10) and caspase-2 (CASP2) that are involved in cell proliferation, DNA methylation, cell cycle, carcinogenesis and apoptosis, respectively [72]. The downregulation of the tumor-suppressive circRHOBTB3 in CRC was also attributed to the excretion of this circular RNA (cirRNA) from cancer cells through exosomes [73]. This process involves sorting by the SNF8 subunit of ESCRT-II. The tumor-suppressive activity of circRHOBTB3 in CRC implies the regulation of metabolic pathways and intracellular reactive oxygen species levels as well as the binding of HuR (Hu-antigen R/ELAV-like protein 1), favoring ubiquitination and degradation of this RNA-binding protein and the downmodulation of the RNA splicing factor PTBP1 (polypyrimidine tract-binding protein 1) [73,74]. Interestingly, antisense oligonucleotides enabling intracellular accumulation of circRHOBTB3 inhibited the proliferation and invasiveness of colon cancer cells in vitro and in tumor growth in nude mice [73].

**Table 2:** Partial list of ncRNAs (miRNAs and lnCRNAs) in exosomes with their biological effects and clinical implications in colorectal cancer.

ncRNA	Producing Cell/Compartment	Recipient Cell	Exosome Isolation	Biological Effect	Clinical Implication	Molecular Target	References
miR-17-5p	Human colon cancer cells (SW480, SW620 cell lines, control epithelial intestinal NCM460 cells)	NA	Human serum from healthy individuals and patients with nonmetastatic and metastatic CRCs; conditioned medium; differential centrifugation	Increased circulating exosomal miR-17-5p in nonmetastatic CRC vs. healthy individuals; higher levels in patients with metastatic CRC	Diagnosis	NA	[75]
miR-19b	Human colon cancer LIM1863, HCT116, and DLD1 cell lines	Human colon cancer HCT116, and DLD1 cell lines	Conditioned medium; ExoQuick Precipitation Kit (System Biosciences)	Stemness, radioresistance, increased tumor growth in nude mice	Radioresistance	Exhausts FBXW7, leading to the activation of the β-catenin signaling pathway	[76]
miR-21	CAFs	Colorectal cancer cells	Primary culture of fibroblasts from human CRCs and control tissue; differential ultracentrifugation	NA	Liver metastasis (orthotopic xenograft)	Known targets: transcripts of PTEN and PDCD4 tumor suppressors	[77]
miR-21	Human colon cancer LS174 cell line	Human colon cancer HT29 and T84 cell lines; human colon FHC cells	Conditioned medium; differential centrifugation	Increased CRC cells proliferation and invasiveness; PDC4 downregulation involved in resistance to 5-FU	Increased proliferation and invasiveness; resistance to 5-FU	Exhausts PDCD4, PTEN and TPM1 transcripts	[78]
miR-21	Colorectal cancer cells (SW480, SW620 and LoVo cell lines)	Liver macrophages/Kupfer cells (membrane-labeled fluorescent EVs injected in mice); THP-1 macrophage cell line	Conditioned medium; filtration, centrifugation	Macrophage polarization into a proinflammatory phenotype (IL-6 release)	Liver metastasis (human liver metastases; orthotopic xenograft in mice)	Binding and activation of TLR7 in liver macrophages; noncanonical miRNA mechanism	[79]
miR-21	Colorectal cells; increased expression from normal epithelium to adenoma and adenocarcinoma	NA	Serum from healthy individuals and patients with colorectal adenomas; ExoQuick kit (System Biosciences, EXOQ20A-1)	Higher level in serum form patients with adenomas vs. healthy individuals	Diagnosis; biomarker for patients with high-risk adenomas	NA	[80]
miR-21-5p	Human colorectal cancer; human colon cancer Lovo, SW620, HT29, SW480, HCT116 and LS174T cell lines	Human endothelial cells (HUVECs)	Serum from CRC patients; conditioned medium; ultracentrifugation	Increased HUVECs proliferation, migration, tubulogenesis	Angiogenesis, vascular permeability in vitro and in vivo (xenografts in nude mice)	Exhausts KRIT1 leading to the activation of the β-catenin signaling pathway, upregulation of VEGFa and Ccnd1	[34]
miR-21-5p	Human colon cancer SW-620 cell line, human colonic epithelial NCM460 cells	Human monocytic leukemia cell line THP-1, murine macrophage line RAW264.7	Plasma; conditioned medium; differential centrifugation	M2 like polarization and PD-L1 expression, resulting in increased PD-L1+CD206+ macrophage abundance and decreased T cell activity; increased tumor growth of mouse CT26.WT cells in syngeneic BALB/c mice	Immunosuppression, inhibition of CD8+ T cell activity	Exhausts PTEN and SOCS1, leading to activation of the PI3K/Akt and STAT1 signaling pathways, respectively	[55]
miR-21-5p	M2 macrophages	Human colon cancer SW48, SW480, and CO-115 cell lines	Conditioned medium; differential centrifugation	Increased proliferation and migration of colon cancer cells	Increased number of lung metastatic nodules (mouse model)	Exhausts transcripts of the transcriptional regulator BRG1	[57]
miR-22-3p	Human bone marrow mesenchymal stem cells (MSCs) transfected with mir-22-3p	Human colon cancer cell lines (Caco-2, SW480, SW620, LoVo and HT29 cell lines) and control colonic epithelial NCM460 cells	Conditioned medium from MSCs overexpressing miR-22-3p; centrifugation/kit extraction	Decreased colon cancer cells proliferation and invasiveness in vitro	Therapeutic approach?	Exhausts RAP2B leading to decreased PI3K levels and p-AKT	[81]
miR-25-3p	Human colorectal cancer cells (SW480, HCT-116 cells)	Endothelial cells (HUVECs), liver and lung endothelial cells	Conditioned medium; differential centrifugation	Increased vascular permeability, angiogenesis	Liver metastasis; high serum exosomal miR-25-3p level in patients with CRC, further increased in patients with metastasis	Silencing of KLF2 and KLF4 leading to decreased expression of ZO-1, occludin and cClaudin-5; increased expression of VEGFR2, p-AKT and p-ERK	[82]
miR-25-3p	Human colorectal cancer cells (HCT-116 cells)	Macrophages	Human serum from healthy individuals and patients with CRC; conditioned medium from HCT-116 cells; Exoquick exosome precipitation solution (System Biosciences); ultracentrifugation	Activation of CXCR4 by CXCL12 increases accumulation of miR-25-3p in exosomes from HCT-116 cancer cells that triggers M2 polarization of macrophages	EMT, invasiveness, angiogenesis, metastasis (experimental) resulting from VEGF release by M2 macrophages	Exhausts PTEN leading to activation of the PI3K/AKT signaling pathway and STAT6 activation	[83]
miR-27b-3p	Human colon cancer LOVO, HCT-116, DLD-1, SW620 and SW480 cells	Endothelial cells (HUVECs)	Conditioned medium; differential centrifugation	Increased blood vessel permeability; increased circulating tumor cells, experimental metastasis; increased level in circulating exosomes from patients with CRC, decreased after tumor resection	Biomarker for CRC metastasis?	Exhausts VE-cadherins	[84]
miR-34a	Murine colon cancer CT-26 cell line	Murine colon cancer CT-26 tumors in Balb/c mice	Conditioned media; Exocib kit (Cibzist fan); loading miR-34a mimic using CaCl2	Decreased tumor growth, prolonged survival of mice, T cell polarization toward CD8+ T subsets among tumor-infiltrating lymphocytes	Cancer nanotherapy; engineered exosomes	NA	[85,86]
miR-92a-3p	Human colon cancer cells (SW480 and SW620 cell lines, control epithelial intestinal NCM460 cells)	NA	Human serum from healthy individuals and patients with nonmetastatic and metastatic CRCs; conditioned culture medium; differential centrifugation	Increased circulating exosomal miR-92a-3p in nonmetastatic colorectal cancer vs. healthy individuals, higher levels in patients with metastatic colorectal cancer	Diagnosis	NA	[75]
miR-92a-3p	Human colon cancer cells (DLD1)	Endothelial cells (HUVEC)	Culture medium; filtration, differential centrifugation	Partial endothelial to mesenchymal transition; increased cell proliferation and loosening intercellular adhesion, which promotes migration	Angiogenesis	Targets the transcripts of claudin-11; integrin subunit alpha 5 (ITGA5), Dickkopf WNT signaling pathway inhibitor 3 (DKK3) and CD69	[87]
miR-92a-3p	CAFs	Colon cancer cells (SW480, SW620 and LoVo cells)	Primary culture of fibroblasts from human colorectal cancer and control tissue; differential ultracentrifugation	Promotes stemness, epithelial-mesenchymal transition (EMT), metastasis and chemotherapy resistance of CRC cells	Liver metastases, resistance to chemotherapies (5-FU/oxaliplatin)	Sponge transcripts of FBXW7 (ubiquitin protein ligase) and MOAP1 (effector of BAX), leading to accumulation of β-catenin transcripts and inhibition of mitochondrial apoptosis	[88]
miR-93-5p	CAFs and control fibroblasts	Human colon cancer HT-29, SW480 and LoVo cell lines; human intestinal epithelial HIEC cells	Conditioned medium; filtration, Ultracentrifugation	Increased growth of SW-480 cells xenografted on nude mice	Radioresistance	Exhausts FoxA1, leading to transcription of TGF-β3 and the activation of TGF-β signaling pathway	[89]
miR-100	Human colon cancer DLD-1 cells and their isogenic derivatives homozygous for wild-type or mutant KRAS	Human colon cancer DLD1 cells	Culture medium; filtration, ultracentrifugation	DLD-1 derivatives expressing wild-type KRAS (DKs-8 cells)	Repression of miR-100 targets in neighboring cells	Involvement of neutral sphingomyelinase in miR-100 sorting in exosomes	[30]
miR-100	Human mesenchymal stem cells	Human colon cancer HCT116 and SW480 cells	Culture medium; ultracentrifugation	Decreased cell proliferation, migration and invasiveness; induction of apoptosis	Therapeutic strategy?	Exhausts mTOR transcripts, leading to downregulation of mTOR, Cyclin D1, K-RAS and HK2, and upregulation of miR-143 and p27	[90]
miR-106b-3p	Human colorectal cancer cells (HCT116, SW480, SNU-C1, SW1116, LoVo and KM12SM)	Colorectal cancer cells	Serum from patients with metastatic or nonmetastatic CRC, Conditioned medium from colonic cell lines. Ultracentrifugation	Induction of EMT, increased experimental metastases; increased level of exosomal miR-106-6p in serum form patients with lung metastasis	Biomarker, therapeutic target	Exhausts deleted in liver cancer-1 (DLC-1)	[91]
miR-106b-5p	Human colorectal cancers; human colon cancer HCT116 and HT29 cell lines	Human monocyte-like THP-1 cells differentiated into macrophages	Serum from patients with CRC; exoRNAeasy Serum/Plasma MaxiKits (QIAGEN, Germany); conditioned medium, ultracentrifugation	M2-like macrophages trigger EMT, facilitating intravasation and liver and lung metastasis of CRC (experimental metastasis)	EMT, metastases	Exhausts PDCD4, leading to PI3Kg/AKT/mTOR signaling pathway activation and M2 macrophage-like polarization	[92]
miR-130b-3p	Human colorectal cancer cells	Macrophages	Human serum from healthy individuals and patients with colorectal cancer; Exoquick exosome precipitation solution (System Biosciences); culture supernatant of HCT-119 cells; ultracentrifugation	Activation of CXCR4 by CXCL12 increases accumulation of miR-130b-3p in exosomes from HCT-116 cancer cells; MiR-130b-3p triggers M2 polarization of macrophages	EMT, invasiveness, angiogenesis and metastasis (experimental) resulting from VEGF release by M2 macrophages	Exhausts PTEN, leading to activation of the PI3K/AKT signaling pathway and STAT6 activation	[83]
miR-141	Human colonic cancer DLD1 (epithelial phenotype), HCT116 and SW620 (mesenchymal phenotype) cell lines	Human MRC5 fibroblast cell line; normal colon fibroblasts	Conditioned medium; differential centrifugation	Exosomes released by epithelial (differentiated) CRC cells are enriched in miR-200 compared with cell lines with a mesenchymal phenotype	Stromal infiltration in CMS4 CRC subtype (mesenchymal)	Suppresses TGF-β-driven fibroblast differentiation into myofibroblast by depleting ZEB1	[31]
miR-146a-5p	Human colon cancer cells HCT-116 overexpressing CXCR7	Mouse CAFs	Human serum from healthy individuals and patients with CRC; conditioned media from human colon cancer HCT-116 cells overexpressing CXCR7; Exoquick exosome precipitation solution (System Biosciences, USA)	Activation of CAFs; induction of EMT and invasiveness of HCT116 and SW620 cell lines	Liver and lung metastasis (experimental mouse models)	Exhausts ZBTB2 transcript, leading to activation of NFkB signaling pathway, secretion of chemokines by CAFs driving EMT of colorectal cancer cells	[93]
miR-146a-5p	Human colon cancer HT-29 and HCT15 cell lines grown as spheroids (stem cell-like)	Colon cancer HT29, HCT15 and CT26 cell lines	Conditioned medium; differential centrifugation	Reprograming into CRC stem cells	Stemness expansion	Promotes stem-like properties and tumorigenicity by targeting Numb in recipient CRC cells	[94]
miR-150	Colonic epithelial cells?	Colon cancer cells	Plasma exosomes; Plasma Exosome Extraction Kits (Thermo Fisher Scientific), cell-culture exosomes; ExoQuick-TC exosome precipitation solution (System Biosciences)	Decreases availability and invasiveness of human colon cancer SW620 cells (in vitro and experimental metastasis)	Downregulation in serum from patients with metastatic colorectal cancer	Targets FTO (α-ketoglutarate dependent dioxygenase/fat mass and obesity-associated gene)	[95]
miR-155-5p	Human colon cancer cells HCT-116 overexpressing CXCR7	Mouse CAFs	Conditioned media from human colon cancer HCT-116 cells overexpressing CXCR7. Exoquick exosome precipitation solution (System Biosciences, USA)	Activation of CAFs; induction of EMT and invasiveness of HCT116 and SW620 cell lines	Liver and lung metastasis (experimental mouse models)	Exhausts SOCS1 transcript leading to activation of JAK/STAT3 signaling pathway, secretion of chemokines	[93]
miR-155-5p	Human M2 macrophages isolated from CRC	Human colon cancer SW48 cells, nontumorigenic CCD 841 CoN cells	Conditioned medium; differential centrifugation	Decreased ZC3H12B accumulation leading to enhanced IL6 transcript stability in cancer cells and inhibition of T cell immune response	IL-6 immune escape	Exhausts ZC3HB12B transcripts	[56]
miR-155-5p	M2 macrophages	Human colon cancer SW48, SW480 and CO-115 cell lines	Conditioned medium; differential centrifugation	Increased proliferation and migration of colon cancer cells	Increased number of lung metastatic nodules (mouse model)	Exhausts transcripts of the transcriptional regulator BRG1	[57]
miR-181a-5p	Colorectal cancer cells	Hepatic stellate cells	Human plasma samples; conditioned medium from HT29, SW480, RKO and SW620 colon cancer cell lines; differential centrifugation	Premetastatic niche formation	Liver metastasis	Sponging SOCS3 leads to inflammatory IL6/STAT3 signaling	[96]
miR-186-5p	M2 macrophages	Human colon cancer SW480 and HCT-8 cell lines	Conditioned medium from THP1 cells differentiated in M0 or M2 macrophages; differential centrifugation	Increased colon cancer cell proliferation and motility	Induction of EMT	Exhausts DLC1, increased activation of β-catenin signaling pathway	[58]
miR-193a	Human colonic cancer SW620 cell line; mouse CT26 cell line		Mouse plasma; differential centrifugation	Inhibits tumor progression (experimental mouse models)	Biomarker prognosis; diagnosis? Increased levels in patients with CRC in a stage dependent manner; therapeutic target?	Exhausted from CRC cells; targets caprin-1, leading to downregulation of Ccnd2 and c-Myc and decreased cell proliferation; MVP favors miR-193a sorting	[71]
miR-200a/b/c	Human colonic cancer DLD1 (epithelial phenotype), HCT116 and SW620 (mesenchymal phenotype) cell lines	Human MRC5 fibroblast cell line; normal colon fibroblasts	Conditioned medium; differential centrifugation	Exosomes released by epithelial (differentiated) CRC cells are enriched in miR-200 compared with cell lines with mesenchymal phenotypes	Stromal infiltration in CMS4 CRC subtype (mesenchymal)	Suppresses TGF-β-driven fibroblast differentiation into myofibroblast by depleting ZEB1	[31]
miR-200a	Human colon cancer SW-620 cell line, human colonic epithelial NCM460 cells	Human monocytic leukemia cell line THP-1, murine macrophage line RAW264.7	Plasma; conditioned medium; differential centrifugations	M2-like polarization and PD-L1 expression, resulting in increased PD-L1+CD206+ macrophage abundance and decreased T cell activity; increased tumor growth of mouse CT26.WT cells in syngeneic BALB/c mice	Immunosuppression, inhibition of CD8+ T cell activity	Exhausts PTEN, leading to activation of the PI3K/Akt signaling pathway	[55]
miR-203	Colon cancer (RKO cells)	Myeloid cells (THP-1 cells)	Human serum from patients with nonmetastatic or metastatic CRCs; conditioned medium from colon cancer cell lines; differential centrifugation	M2 macrophage polarization; high serum exosomal miR-203 associated with poor prognosis; conversely, high miR-203 in tumor tissue is associated with a better prognosis; promotes experimental metastases of RKO cells; in vitro proliferation, invasiveness and motility of cancer cells unaffected	Liver metastasis	NA	[97]
miR-204-5p	HEK293T cells stably expressing miR-204-5p	Human colon cancer LoVo and HCT116 cells	Conditioned medium; differential centrifugation	Decreases cell proliferation and colony formation of CRC cells, induction of apoptosis, sensitization to oxaliplatin in vitro and in vivo (mouse models)	Cancer nanotherapy	miR-204-5p exhausts RAB22A and Bcl2	[98]
miR-208b	Human colon cancer SW-480 cell line and oxaliplatin-resistant derivatives; human colon NCM-460 cell line	Mouse CD4+ T lymphocytes	Serum from patients with CRC; conditioned medium from cell lines; gradient centrifugation	Increased growth of mouse CT-26 tumors in syngeneic Balb/c mice	Oxaliplatin resistance; putative biomarker of resistance	Exhausts PDCD4, leading to Treg expansion	[60]
miR-210	Human colon cancer HCT-8 cells (subpopulation growing in suspension)	Human colon cancer HCT-8 cells	Conditioned medium; Exosome Precipitation Solution (Macherey-Nagel)	Promotes EMT and resistance to anoikis	Oxaliplatin and 5-FU resistance	NA	[99]
miR-217	Colorectal cancer cells	NA	Serum from patients with colorectal tumors; conditioned medium from human colon cancer HT-29, SW480, HCT-116, SW620, LoVo, SW48, DLD-1, Caco2 and HT-15 cells, and human colonic epithelial NCM460 cells. ExoQuick kit (SBI, USA)	Decreased exosomal miR-217 level in serum from patients with CRC compared to patients with adenoma and healthy individuals; increased level following chemotherapy	Diagnosis, prognosis	NA	[100]
miR-221	Colon cancer (SW480 cells)	Liver stromal cells	Serum; exosome isolation kit (Invitrogen); conditioned medium; differential centrifugation	NA	Liver metastases; decreased overall survival	Exhausts SPINT1 transcripts, leading to hepatocyte growth factor activation and liver metastatic niche formation	[101]
miR-221-3p	Colon cancer cells (HCT116 and Caco-2 cell lines)	Human endothelial cells (HUVECs)	Conditioned medium from HCT116 and Caco-2 cells; centrifugation; ExoQuick™ Exosome Precipitation Solution (System Biosciences)	Increased proliferation and motility of endothelial cells in vitro	Angiogenesis	Exhausts SOCS3, leading to STAT3 signaling pathway and upregulation of VEGFR2	[33]
miR-222	Colon cancer (SW480 cells)	Liver stromal cells	Serum exosomes: exosome isolation kit (Invitrogen); colon cancer cell lines. Derived exosomes: differential centrifugations	NA	Liver metastases; decreased overall survival	Exhausts SPINT1 transcripts, leading to hepatocyte growth factor activation and liver metastatic niche formation	[101]
miR-224-5p	Human colon cancer SW-620 cells	Human colon CCD 841 CoN and colon cancer SW-620 cell lines	Conditioned medium; GETTM Exosome Isolation Kit (GeneExosome technologies).	Increased viability, proliferation, migration and invasiveness	Increased growth of SW-620 cells xenografted in nude mice	Exhausts CMTM4 (CKLF-like MARVEL Transmembrane Domain Containing 4)	[27]
miR-224-5p	CAFs	Human colon cancer CT116, SW480, Caco-2, LoVo and T84 cells and control colonic epithelial NCM-460 cell line	Conditioned medium from CAFs; differential centrifugation	Promotes proliferation, migration, invasiveness and antiapoptotic abilities of CRC cells	miR-224-5p overexpressed in CRC and in CAFs	Exhausts SLC4A4	[102]
mir-320c	Human colorectal cancer, human colon cancer HT-29 and HCT-116 cell lines	NA	Plasma from healthy individuals and patients with CRC; conditioned medium; ExoEasy Plasma (QIAGEN)	Enrichment in Evs	Diagnosis, follow-up; reprogramming metastasized cells into a metastasis-favorable mesenchymal-epithelial transition state?	NA	[103]
miR-335-5p	Human colon cancer SW620 cells	Human colon cancer SW480 cells	Conditioned medium from human colon cancer SW480 and SW620 cells; ultracentrifugation	Transfer EMT phenotype; increased metastatic ability in vivo	EMT; metastasis	Exhausts RASA1 (GTPase-activating protein)	[104]
miR-424	Human CRC cell lines HT116, HT29, DLD-1, HCT-8, Caco-2, WiDr and SW480 and mouse CRC cell lines CT26 and MC38	Human primary T cells; primary human dendritic cells from PBMCs	Conditioned medium; density gradient centrifugation	Hypoxia up-regulates miR-424 and enhances EV production	Resistance immune checkpoint blockade; putative therapeutic target	Exhausts CD28 and CD80 expression levels in T cells and dendritic cells	[52]
miR-425-5p	Human colon cancer HCT116 and SW620 cells	Macrophages (murine macrophages RAW264.7 and human monocytic leukemia cells THP-1)	Human serum from healthy individuals and patients with colorectal cancer; Exoquick exosome precipitation solution (System Biosciences); conditioned medium; ultracentrifugation	Activation of CXCR4 by CXCL12 increases accumulation of miR-425-5p in exosomes from HCT-116 cancer cells that triggers M2 polarization of macrophages	EMT, invasiveness, angiogenesis and metastasis (experimental) resulting from VEGF release by M2 macrophages	Exhausts PTEN, leading to activation of the PI3K/AKT signaling pathway and STAT6 activation	[83]
miR-548c-5p	Human colon cancer HCT116 and SW480 cells	Human colon cancer HCT116 and SW480 cells	Conditioned medium; Exosome Isolation and Purification Kit (Umibio)	Decreased cell proliferation, migration and invasiveness	Low levels associated with a poor prognosis; biomarker prognosis?	Decreased miR-548c-5p in serum exosomes from patients with CRC; exhausts HIF1 transcripts leading to CDC42 downmodulation	[105,106]
miR-590-3p	CAFs	Human colon cancer SW480, SW620, HCT116, LOVO, HT29 and SW116 cell lines and control colon epithelialNCM-460 cells	Conditioned medium; filtration; ExoQuick Exosome Precipitation Kit (System Biosciences)	Enhanced resistance of CRC cells to radiotherapy in vitro and in vivo (xenografts in nude mice)	Radioresistance; putative biomarker of CRC and of response to radiotherapy	Exhausts CLCA4, leading to activation of the PI3K/AKT signaling pathways	[107]
miR-934	Colorectal cancer cells	M2 macrophages, Kupffer cells	Human serum; centrifugation	Premetastatic niche formation	Liver metastases; decreased overall survival (OS) and disease-free survival	PTEN downregulation, activation of PI3K/AKT signaling pathway; CXCL13 secreted by recipient cells triggers invasiveness of colorectal cancer cells	[108]
miR-1229	Human colorectal cancer, human colon cancer HCT-116 cells	Human umbilical vein endothelial cells (HUVECs)	Serum, conditioned medium; filtration, ultracentrifugation	Increased proliferation, migration and tubulogenesis of HUVECs	Angiogenesis; high circulating miR-1229 associated with poor overall survival in CRC patients	Exhausts HIPK2 (Homeodomain Interacting Protein Kinase 2), promoting transcriptional activity of MEF2C and VEGF accumulation	[47]
miR-1246	Human colon cancer DLD-1, WiDr, SW480 and COLO201 cell lines	Human umbilical vein endothelial cells (HUVECs)	Conditioned medium; filtration, ultracentrifugation	Increased proliferation, migration and tubulogenesis of HUVECs	Angiogenesis	Enriched in circulating EVs; exhausts PML (promyelocytic leukemia) protein, leading to activation of Smad 1/5/8 signaling in HUVECs	[48]
miR-6869-5p	Human colorectal cancers	Human colon cancer colo-205 and HCT-116 cells	Serum from patients with CRC; Total exosome isolation kit (Invitrogen)	Decreased cell proliferation, promotes apoptosis in vitro; does not affect cell migration in vitro; enhanced tumor growth in vivo (xenograft in nude mice)	Low levels associated with poor prognoses; biomarker prognosis?	Downregulated in colorectal tumors and CRC-derived exosomes; exhausts TLR4 and downmodulates NFkB signaling pathway	[105,109]
miR-8073	Human colon cancer HCT116 and HT-29 cell lines	NA	Conditioned medium; differential centrifugation	Inhibits proliferation of cancer cells but not of normal HMVEC cells (microvascular endothelium); decreased tumor growth (experimental mouse model)	Therapeutic strategy?	Tumor suppressor miRNA exhausted from cancer cells; targets OXM1, CASP2, MBD3, KLK10 and CCND1	[72]
PGM5-AS1 (PGM5 antisense RNA 1)	HEK293T cells overexpressing PGM5-AS1	Human colon cancer DLD1 cell derivative resistant to oxaliplatin	Conditioned medium; differential centrifugation; oxaliplatin loading by electroporation	Inhibition of proliferation, metastasis and acquired oxaliplatin resistance of colon cancer cells in vivo; reversion of drug resistance	Cancer nanotechnology; engineered exosomes	PGM5-AS1 upregulates the nucleoside diphosphate kinase NME1 by sponging hsa-miR-423-5p, and it downregulates PAEP (member of the kernel lipocalin superfamily) by recruiting SRSF3 to promote alternative splicing	[110]
lncRNA CCAL	CAFs, tumor stroma	Human colon cancer cells (SW480, HCT-116 cells)	Conditioned medium of control fibroblasts and CAF primary cultures; differential centrifugation	Activation of Wnt pathway	Oxaliplatin resistance in vitro and in vivo	Interaction with the RNA binding protein HuR leads to stabilization of β-catenin transcript and activation of the Wnt pathway	[111]
lncRNA CRNDE-h	Colorectal cancer cells	NA	Serum from patients with colorectal tumors; conditioned medium from human colon cancer HCT116, SW620, SW480, HT29 and LoVo cells and human colonic epithelial FHC cells; ExoQuick kit (SBI, USA)	Gradual increased levels of exosomal CRNDE-h in serum from patients with adenoma to adenocarcinoma; associated with poor prognoses	Diagnosis, prognosis	NA	[112]
lncRNA CRNDE-p	Human colorectal cancer HT-29, SW480, HCT-116, SW620, LoVo, SW48, DLD-1, Caco2 and HT-15 cell lines and human colonic epithelial NCM460 cells	NA	Serum from patients with colorectal tumors; conditioned medium from human colonic cell lines; ExoQuick kit (SBI, USA)	Increased levels of exosomal CRNDE-h in serum from patients with adenocarcinoma compared to patients with adenomas and healthy individuals; associated with poor prognoses	Diagnosis, prognosis	NA	[100]
lncRNA H19	CAFs	Human colon cancer SW480 and HCT116 cell lines	Conditioned medium from primary control fibroblasts and CAFs of CRC patients; differential centrifugation	Activation of Wnt pathway; stemness	Resistance to oxaliplatin in vitro and in vivo	Sponges miR-141 that exhausts β-catenin	[113]
LncRNA LINC00659	CAFs	Human colon cancer LOVO and SW48 cell lines	Conditioned medium from primary control fibroblasts and CAFs of CRC patients; differential centrifugation		Increased proliferation, migration, invasion and EMT	Sponges miR-342-3p that exhausts ANXA2	[114]
lncRNA MALAT1	Human colon cancer LoVo, HCT-8, SW620 and SW480 cell lines	Colorectal cancer cells	Conditioned medium from human colon cancer cells; differential centrifugation	Increased proliferation, migration and invasiveness; increased metastatic properties (experimental)	Diagnostic biomarker? Therapeutic target?	Sponges miR-26a/26b leading to upregulation of fucosyltransferase 4 (FUT4); activation of the PI3K pathway	[115]
LncRNA RPPH1	Human colon cancer HCT8, SW620 and HT-29 cell lines	Human monocyte-derived macrophages	Conditioned medium; differential centrifugation	Macrophage M2 polarization; increased tumor growth and metastasis (experimental)	Metastasis, biomarker?	NA	[116]
lncRNA SNHG10	Human colon cancer SW480 cell line	Human natural killer NK92-MI cell line	Conditioned medium; differential centrifugation	Decreased proliferation, viability and cytotoxicity (production of IFN-γ, perforin and granzyme B) of NK cells. Increased growth of SW480 cells xenografted in nude mice	Immunosuppression of NK cells	lncRNA SNHG10 promotes the accumulation of INHBC (Inhibin Subunit Beta C) in NK cells, a member of the TGF-β superfamily	[53]
lncRNA UCA1 (urothelial carcinoma-associated 1)	Cetuximab-resistant Caco2-CR cells	Parental human colon cancer Caco-2 cells	Serum from patients with colorectal cancer; centrifugation; conditioned medium from Caco-2 cells; ExoQuick TC kit (SBI)	Resistance to cetuximab in vitro and in vivo (experimental); transfer of resistance to sensitive cells	Resistance cetuximab, biomarker response treatment	Sponges miR-495 that exhausts the MET receptor tyrosine kinase and its ligand HGF; LncRNA UCA1 proved also to promote 5-FU resistance by sponging miR-204-5p, leading to the activation of the CREB1/BCL2/RAB22A axis	[117,118,119]
lncRNA UCA1 (urothelial carcinoma-associated 1)	Colorectal cancer cells	Human colon cancer CT116, DLD1, SW480, RKO and HT-29 cell lines	Upregulated in CRC. Increased proliferation, migration and invasiveness of colonic cell in vitro; enhanced metastatic potential (experimental)	Upregulated in CRC; increased proliferation in vitro and in vivo	Metastasis?	Sponges miR-143, which exhausts MYO6	[120]
lncRNA WEE2-AS1	CAFs	Colorectal cancer cells (HCT 116, HT-29 cells); AOM/DSS experimental carcinogenesis	Conditioned medium from human CAFs and control fibroblasts; plasma from patients with CRC; differential centrifugation	Inhibition of Hippo pathway	Increased cancer cell proliferation in vitro; promotes experimental carcinogenesis in mouse; High level in plasma of patients with CRC associated with poor prognoses	Increased MOB1A proteasomal degradation by enhancing its binding to the E3 ligase praja2.	[121]
circ_0000338	Human colon cancer cells (human colon cancer SW-480 and HCT-116 cells)	Colon cancer cells	Serum from patients with CRC; conditioned medium; differential centrifugation	Upregulated in human colorectal cancers	Resistance to 5-FU, transfer of resistance to sensitive cells	Sponges miR-217 and miR-485-3p	[122]
circ-ABCC1 (hsa_circ_0000677)	CD133+, CD133-Caco-2 and HCT15 colon cancer cells	Caco-2 and HCT15 colon cancer cells	Conditioned medium of the human colon cancer Caco-2 and HCT-15 cells; ExoQuick precipitation reagent (Invitrogen)	Activation of Wnt signaling pathway	Cancer cell stemness	Interaction and translocation of β-catenin to the nucleus	[123]
circ-FBXW7	FHC cells, engineered exosomes	Human colon cancer SW-480 and HCT-116 cell derivatives resistant to oxaliplatin	Conditioned medium from FHC cells; ultracentrifugation; electroporation of circ-FBXW7 into exosomes	Restauration of sensitivity to oxaliplatin	Cancer nanotherapy; engineered exosomes	Sponges miR-18b-5p	[124]
circLPAR1	Normal colonic epithelial cells	Colorectal cancer cells	Plasma from patients with colorectal cancer; ExoQuick Plasma Prep with Thrombin kit (SBI, USA); conditioned medium from human colonic epithelial cells FHC cells and colon cancer HCT116 and DLD1 cells; ExoQuick TC kit (SBI, USA)	Decreased during CRC development, normalized after tumor resection	Diagnosis	circLPAR1 binds eIF3h and suppresses the METTL3-eIF3h interaction, decreasing the translation of oncogene BRD4	[125]
circN4BP2L2	CAFs	Human colon cancer cells (Lovo cell line)	Conditioned medium from CAFs and control fibroblasts; differential centrifugation	Increased proliferation and migration in vitro; increased tumor growth and experimental metastases	Liver metastasis (experimental)	Sponges miR-664b-3p, which exhausts HMGB3 (high mobility group box 3) involved in Wnt signaling	[126]
circN4BP2L2	CAFs	Human colon cancer cells (Lovo cell line)	Conditioned medium from CAFs and control fibroblasts; differential centrifugation	Increased cell proliferation, decreased apoptosis	Cancer cells stemness and oxaliplatin resistance	Interacts with the RNA binding protein EIF4A3, leading to its upregulation and activation of the PI3K/AKT signaling pathway	[127]
circPABPC1	Colon cancer cells	NA	Plasma from patients with colorectal cancer; ExoQuick Exosome Precipitation Solution (SBI); conditioned medium of the human colon cancer Caco-2, SW60 and LoVo cells	Recruitment of KDM4C lysine demethylase to the promoter of the transcriptional regulating factor HMGA2, leading to upregulation of effectors of EMT; protection of ADAM19 and BMP4 transcripts from miR-874/miR-1292; decreased colon cancer SW620 and Lovo cell proliferation, invasion and migration in vitro and in vivo (xenograft growth of SW480 cells and experimental liver metastasis)	Liver metastasis		[128]
circRHOBTB3	Human colon cancer RKO, SW480, HCT8 and HCT116 cells and NCM4060 colonic epithelial cells	NA	Serum from healthy individuals and patients with colorectal cancers; conditioned medium; ultracentrifugation	Tumor-suppressive role in CRC, excreted out of cells to sustain cancer cell fitness; intracellular accumulation inhibits EMT, cell proliferation and invasion	Therapeutic strategy?	Decreased circRHOBTB3 accumulation in tumor samples, increased in circulating exosomes	[73]

NA: not applicable.

## 3. Role of Extracellular Vesicles on Premetastatic Niche Formation

The preferential ability of breast cancer to metastasize independently of anatomical considerations in brain, lung and bone led Stephen Paget in 1889 to put forward the seed-and-soil hypothesis. This assertion proposes a critical role for the microenvironment of target organs in enabling the implantation and growth of cancer cells, and it is further supported, for instance, by the spread of lung metastases to the brain and the tropism of prostate metastases in bone. Chemokines secreted by cancer cells might shape microenvironments in target tissues, whereas remote tissues might release chemo-attractants, thus guiding metastasis [129]. More recently, the release of extracellular vesicles by cancer cells has provided new insights into Paget’s assumption.

Regarding colorectal cancer, vascularization through the portal system as well as lower rectum drainage through the internal iliac vein might partly explain the preferential metastatic spread of colorectal cancer in liver tissue and the higher rate of lower rectum metastasis in lung tissue [130]. In this connection, phylogenetic analysis of lymph node and liver metastases of CRCs revealed that two-thirds of these metastases originate from independent subclones in the primary tumor. This suggests that liver metastases are preferentially seeded hematogenously [131]. Accordingly, colorectal cancer cells that disseminate through lymph nodes might reach the venous circulation through the left subclavian vein leading to the lung. The fact that lymph node status is an important prognostic factor in the staging of CRCs might reflect the overall propensity of some primary tumors to metastasize, with local dissemination being more efficient than distant implantation [131].

Nevertheless, CRC-derived EVs could exert a critical role in facilitating target tissue remodeling and premetastatic niche formation through the activation/reprogramming of fibroblasts, epithelial, immune and endothelial cells. Hoshino et al. reported that the proteomic signature of tumor exosomes allows a preferential uptake by selective remote cells, and their reprogramming and the formation of a premetastatic niche contributes to metastatic organ tropism [132]. The proteomic and biodistribution analyses of exosomes from cancer cell lines of different tissue origins revealed a critical role of exosomal integrins in addressing organ-specific colonization: exosomal integrins α6β4 and α6β1 were associated with lung metastasis, while exosomal integrin αvβ5 was linked to liver metastasis [132].

### 3.1. EV Protein Cargo in Premetastatic Niche Formation

Profiling of exosomes from patients with colorectal cancers compared to healthy individuals with mass spectrometry analysis allowed the identification of 36 upregulated proteins and 22 downregulated proteins [133]. The upregulated proteins, including MMP9 (matrix metalloproteinase-9), ADAMTS13 (ADAM metallopeptidase with thrombospondin type 1 motif 13) and CRP (C-Reactive Protein), are known to be involved in extracellular matrix remodeling, vascular permeability and tumor-promoting inflammation. Interestingly, the downregulated proteins were IGF1 and members of the HSP family that favor CRC cell survival. This suggests the existence of not only exosomes with distinct protein contents acting as paracrine/autocrine factors to sustain cell survival and proliferation during the development of colorectal cancer, but also exosomes released into the circulation for establishment of the premetastatic niche [133]. In this connection, the exosomes of the human colon cancer SW620 cell line that originate from lymph node metastasis are enriched in S100A8, HGF receptor MET and signal transduction molecules (Ephrin-B2, EGFR, protein jagged-1, SRC) compared with the isogenic SW480 cell line derived from the corresponding primary tumor [134].

Other proteins proved to be enriched in exosomes and were thereby associated with human colorectal cancer metastasis. High levels of integrin beta-like 1 (ITGBL1) in the primary tumors and high expression in extracellular vesicles were linked with metastasis and decreased overall survival [42]. The ITGBL1 gene is overexpressed with other genes related to cell adhesion and metastasis (fibronectin 1, collagen, type VIII, alpha 2, matrix metalloprotein 9 and chemokine CXCL12) under the control of the RUNX2 transcription factor. Biodistribution analysis of ITGBL1-rich vesicles in mouse models revealed their tropism for hepatic stellate cells as well as for myofibroblasts and macrophages in liver and lung tissue but not for endothelial cells. Furthermore, these vesicles enhanced the growth of liver and lung metastases in experimental mouse models. Exosomal ITGBL1 facilitates premetastatic niche formation by interacting with tumor necrosis factor alpha-induced protein 3 (TNFAIP3) in fibroblasts and stellate cells, leading to the stimulation of the NF-κB signaling pathway. The corresponding activated fibroblasts release the pro-inflammatory cytokines IL-6 and IL-8, which promote stemness, aggressiveness and EMT of the human colon cancer HCT116 cell line in vivo and in vitro [42]. Interestingly, the traditional Chinese herbal JianPi JieDu recipe downregulates ITGBL1 in vesicles released by the human colon cancer LoVo cell line, impeding fibroblast activation in vitro and in vivo in experimental metastasis [135]. Lymph node metastasis in patients with colorectal cancer is associated with enriched exosomal IRF-2 (interferon regulatory factor 2) in serum [41]. Using the mouse colon carcinoma CT-26 cell line, Sun et al. demonstrated that the engulfment of IRF2-rich vesicles by macrophages induces VEGF-C release, triggering lymphangiogenesis and lymph node metastasis [41]. Exosomes produced by the human colon cancer HCT116, SW620, HT29 and SW480 cell lines contain high levels of the nucleolar protein HSPC111 (nucleolar protein 16). The uptake of HSPC111 by hepatic stellate cells causes their reprogramming into cancer-associated fibroblasts (CAFs) and the expression and secretion of CCL5, which further sustains exosomal HSPC111 excretion from cancer cells; thus, it creates a positive feedback loop and triggers EMT of colon cancer cells in vitro and experimental metastasis in vivo. Mechanistically, HSPC111 interacts with ATP citrate lyase (ACLY), which leads to increased acetyl-CoA levels, enhanced histone H3 acetylation and epigenetic regulation of gene transcription [38]. In line with these experimental observations, HSPC111 levels was found to be higher in serum exosomes from patients with metastatic colorectal cancer compared with patients with nonmetastatic CRC and with healthy individuals. The human antigen R (HuR) is overexpressed in colorectal cancer, and it is associated with lung metastasis and poor survival [39]. HuR vesicles might initiate the remodeling of bronchial epithelium, facilitating colon cancer implantation [39]. This RNA binding protein stabilizes tumor-promoting mRNAs by binding to 3’UTR U-rich elements. HuR-containing exosomes derived from colon cancer cells are up-taken by the human nontumorigenic lung epithelial BEAS-2B cells, which promote their migration and proliferation through the stabilization c-MYC transcripts and a decreased accumulation of the CDK inhibitor p21. 

On the other hand, Angiopoietin-like protein 1 (ANGPTL1), which is known to exert metastatic suppressor activity in several cancers, is downregulated in vesicles derived from human colorectal cancers. In experimental mouse models, exosomes containing ANGPTL1 protein curtail liver metastasis and impede vascular leakiness in the liver premetastatic niche. The uptake of ANGPTL1 by Kupffer cells inhibits the JAK2-STAT3 signaling pathway, leading to decreased MMP9 expression, which prevents liver vascular permeability [36].

### 3.2. EV ncRNA Cargo in Premetastatic Niche Formation

Besides proteome, ncRNAs delivered by exosomes also contribute by modifying the microenvironments of target tissues, performing premetastatic niche priming and facilitating colonization by circulating cancer cells (Table 2). The relative accumulation of miR-181a-5p in serum EVs was markedly higher in patients with metastatic colorectal cancer compared to patients with tumors at stage I–II [96]. Similarly, the highly metastatic human colon cancer SW620 and RKO cell lines released more miR-181a-5p-rich extracellular vesicles compared to the poorly metastatic HT29 and SW480 cells. This enrichment is favored by the FUS RNA binding protein that mediates packaging of miR-181a-5p into extracellular vesicles [96]. The uptake of miR-181a-5p activates hepatic stellate cells by depleting SOCS3 (suppressor of cytokine signaling 3) transcripts, triggering the inflammatory IL-6/STAT3 signaling pathway. In vitro and in vivo experiments revealed that activated hepatic stellate cells shape liver premetastatic niches by remodeling ECM through increased expression of α-smooth actin and fibronectin and reduced expression of vitronectin and tenascin C. Furthermore, these cells secrete the chemokine CCL20, which acts as a chemoattractant for colorectal cancer cells overexpressing the CCL20 receptor CCR6, and it induces a ERK1/2/Elk-1/miR-181a-5p positive feedback loop [96].

Exosomal miRNA from colorectal cancer cells could also promote premetastatic niche formation by inducing M2 macrophage polarization (Table 2). MiR-934 was identified as a highly abundant miRNA in metastatic colorectal cancer, and high miR-934 expression in serum exosomes was correlated with liver metastases [108]. MiR-934 is packaged in EVs with the hnRNPA2B1 RNA-binding protein, and it causes M2 macrophage polarization in vitro by exhausting PTEN transcripts [108], resulting in PI3K/AKT signaling pathway activation [136,137]. Exosomal miR-934 triggers the secretion of CXCL13 with M2 macrophages and Kupffer cells, which enhances the migration and invasiveness of the human colon cancer SW480 and RKO cells as well as metastasis in nude mice. Moreover, CXCL13 activates the NFκB pathway in CRC cells and upregulates MMP2, MMP9 and miR-934 [108]. Similarly, miR-203 in serum exosomes from patients with colorectal cancer promotes monocyte differentiation towards M2 macrophage phenotypes [97]. High expression of exosomal miR-203 is associated with liver metastasis and poor prognoses. In experimental mouse models of liver metastasis, overexpression of miR-203 in RKO colon cancer cells facilitates tumor implantation, which is further potentiated by coinjection of THP1 monocytes. This suggests that miR-203-induced monocyte differentiation to M2-tumor-associated macrophages favors the formation of premetastatic niches. In contrast, miR-203 does not affect cancer cell proliferation, invasiveness or migration in vitro [97]. MiR-106-5p cargo in CRC EVs depletes PDCD4 (Programmed Cell Death 4), leading to the stimulation of the PI3K/AKT/mTOR signaling pathway and M2 macrophage-like polarization. In turn, these M2 macrophages trigger the EMT of colon cancer cells, favoring intravasation and lung and liver metastases in experimental mouse models [92]. The activation of colorectal cancer cells by CXCL12 might contribute to the metastatic process through two pathways involving the crosstalk with fibroblasts and macrophages. CXCL12-activated CRC cells recruit macrophages to the invasive front of the tumor, and they induce their M2 polarization by transferring via exosomes a panel of miRNAs (including miR-25-3p, miR-130b-3p and miR-425-5p) that deplete PTEN transcripts and activate STAT6. In turn, these M2 macrophages release VEGF, IL-10 and IL-4, which promote the EMT of cancer cells as well as angiogenesis and liver metastasis [83]. On the other hand, the uptake of miR-146a-5p and miR-155-5p from CRC cell-derived exosomes activates CAFs through JAK2-STAT3/NF-κB signaling by targeting ZBTB2 (zinc finger and BTB domain containing 2) and SOCS1 (suppressor of cytokine signaling 1), respectively. Reciprocally, the subsequent release of inflammatory cytokines (IL-6, TNF-α, TGFβ and CXCL12) from CAFs causes EMT and a pro-metastatic switch of CRC cells, and it also facilitates tumor formation and lung metastasis [93]. High levels of exosomal miR-221/222 in the serum of patients with metastatic colorectal cancer are associated with poor overall survival. These miRNAs exhaust the transcripts of SPINT1, a serine protease inhibitor for HGF activator, which leads to HGF activation in liver stromal cells and liver microenvironment remodeling [101]. Furthermore, activated HGF might support the spread of metastatic colorectal cancer cells because they overexpress the HGF receptor MET [138].

Colorectal cancer-derived exosomes also drive premetastatic niche formation through the activation of endothelial cells. The uptake of exosomal miR-25-3p in endothelial cells stimulates vascular permeability and angiogenesis by targeting KLF2 (Krüppel-like factor 2) and KLF4 (Krüppel-like factor 4) transcription factors. KLF2 and KLF4 silencing downregulates ZO-1, occludin and claudin-5 expression and increases VEGFR2 expression. The premetastatic niche formation as a result of exosomal miR-25-3p-mediated vascular leakiness was validated in an experimental model of metastasis, and it is consistent with the upregulation of miR-25-3p associated with metastatic human CRCs [82].

### 3.3. Stroma-Derived EVs in Premetastatic Niche Formation

Tumor stroma-derived extracellular vesicles also exert a critical role in the metastatic process. MiR-21 accumulation in CAF EVs is correlated with CRC progression, especially with liver metastasis. In an experimental mouse model, the cointracecal injection of fibroblasts overexpressing miR-21 with the human colon cancer SW620 cells results in a greater number and size of liver metastatic tumors, highlighting the significance of CAF-derived exosomes in the metastatic cascade [77]. It should be noted that miR-21 targets the transcripts encoding the PTEN and PDCD4 tumor suppressors. Interestingly, miR-21-enriched extracellular vesicles also proved to sustain liver inflammatory premetastatic niches through macrophage polarization with a noncanonical miRNA mechanism involving miR-21 binding and activation of TLR7, leading to the release of IL-6 [79].

## 4. Extracellular Vesicles in Colorectal Cancer Diagnosis and Follow-Up

Liquid biopsies are less invasive than tissue biopsies and enable the safe and serial collection of samples for diagnostic purposes or for patient follow-up at an affordable cost. In this concern, there is a growing interest in EVs from biological fluids as potential cancer biomarkers. Urinary extracellular vesicles may constitute suitable biomarkers for renal, bladder and prostate cancers [139]; salivary extracellular vesicles might allow detection and following of head, neck and esophageal carcinomas [140,141], whereas blood-derived EVs might be accurate for the early diagnosis, prognosis and prediction of therapy responses for many types of cancers, including breast, lung, liver stomach, brain, cervix and ovarian cancers [142,143,144,145,146,147,148,149]. Accordingly, the molecular composition of extracellular vesicles mirrors the functional status and activity of the parental cells which produced them, but it is also rich in specific biomolecules related to cellular transformation, allowing consideration of their use in the noninvasive diagnosis and follow-up of a wide range of tumors, including colorectal cancer.

### 4.1. EV Lipid Cargo as Biomarker for the Diagnosis and Prognosis of CRC

So far, lipidome analysis of plasma extracellular vesicles does not provide a clear biomarker for the diagnosis of colorectal cancer. A decrease in fatty acids in saturation and a shift of 34:1 phosphatidylcholine (PC), phosphatidylinositol (PI) and phosphatidylethanolamine (PE) for healthy individuals to 38:4 species for people with colonic lesions was shown; the ratio of these species shows diagnostic potential [150]. Nevertheless, Elmallah et al. identified an increased accumulation of PC 34:1 species in serum EVs from patients with nonmetastatic colorectal cancer compared to patients with metastases and healthy individuals [151].

### 4.2. EV Protein Cargo as Biomarker for the Diagnostic and Prognostic of CRC

Regarding proteome, mass spectrometry analysis of extracellular vesicles has enabled potential biomarkers of CRC to be found. A proof of concept of the ability of EVs to identify individuals with cancer and to establish its tissue origin was recently performed using a proteomic approach coupled with machine learning [152]. Proteome profiling was performed on extracellular vesicles isolated from the tissues and plasma of 497 normal and cancer samples and characterized as Exo S, Exo L and exomeres. This analysis allowed prediction of their discriminatory value with a sensitivity of 100% and a specificity of 92% to distinguish between individuals with and those without cancer. Furthermore, the profiling of tissue-derived extracellular vesicles enabled discrimination of melanoma, colorectal, pancreatic and lung cancers [152]. In a study encompassing 100 individuals equally distributed as healthy individuals, patients with early/late adenomas and patients with adenocarcinomas from stage-I to stage-IV, liquid chromatography–tandem mass spectrometry of serum extracellular vesicles identified six proteins, GCLM (involved in glutathione synthesis), KEL (endopeptidase), APOF (apolipoprotein F), CFB (complement factor B), PDE5A (cGMP-specific phosphodiesterase) and ATIC (purine biosynthetic pathway), that distinguished healthy control, early neoplasia and advanced neoplasia patients from each other [153].

Other studies reported higher levels of EVs containing glycosylated fibrinogen beta chain (FGB) and beta-2-glycoprotein 1 (β2-GP1) in plasma from patients with colorectal cancer compared to a control group. Furthermore, these markers achieved higher sensitivity and specificity for the diagnosis of CRC compared with carcinoembryonic antigen (CEA) and carbohydrate antigen 19-9 (CA19-9), and thus, they might constitute biomarkers for diagnosing patients with early-stage CRC [154]. Similarly, based on data mining of candidate proteins and proteome analysis of EVs isolated from sera of patients with colorectal cancer and healthy individuals, Shiromizu et al. demonstrated that annexins A3, A4 and A11 extracellular vesicle-derived peptides detect stage II CRC with a greater sensitivity than CEA [155]. The level of EVs containing SPARC (extracellular matrix) and LRG1 (cell signaling) are higher in the serum from patients with stage III colon cancer than in the serum from healthy control individuals, and they were predictive of disease recurrence. Interestingly, the increased accumulation of SPARC and LGR1 in EVs seems to be relatively selective of colon cancer because it was not observed in patients with gastric, thyroid or cervix cancer [156]. Importantly, SPARC was previously reported to be ectopically expressed in the stroma of digestive tumors but not by cancer cells themselves [157,158]. Of note, a decrease of QSOX1 (Quiescin Sulfhydryl Oxidase 1) containing EVs originating from CAFs was found in the sera of patients with CRC [159].

After the purification of extracellular vesicles from plasma and a data-independent acquisition mass spectrometry (DIA-MS) analysis of the samples, Xi Zheng et al. found that phosphorylated fibronectin 1, haptoglobin, calgranulin-B and fibrinogen α chain were significantly associated with cancer progression from healthy individuals to patients with colonic adenoma and adenocarcinoma, with fibrinogen α chain being the most distinguishing biomarker [160].

Analysis of EVs from stage II/III CRCs and adjacent tissues revealed poorer immunity and chronic inflammatory responses in the group of patients who relapsed, and it identified HLA-DPA1 (HLA class II histocompatibility antigen, DP alpha 1 chain), S100P (protein S100-P), NUP205 (Nuclear pore complex protein Nup205) and PCNA (proliferating cell nuclear antigen) expression in adjacent tissue as associated with the risk of disease recurrence [161].

Extracellular vesicles might also allow prediction of the clinical outcomes in patients with metastatic colorectal cancer. Accordingly, elevated blood concentrations of total extracellular vesicles and CD133+ (transmembrane protein expressed in stem cells) EVs before treatment are correlated with shorter overall survival of patients with metastatic colorectal cancer. Furthermore, higher CD133+ EV concentrations are associated with a lower overall response rate to first-line systemic therapy, and high concentrations might serve as biomarkers to improve risk stratification and to optimize treatment strategies in metastatic cancer [162]. Chemokine ligand 7 (CXCL7)-enriched EVs are linked with metastatic CRCs. The level of CXCL7 EVs was found as a biomarker of early response in patients with liver metastasis receiving systemic chemotherapy that dropped down after secondary tumor resection, suggesting metastatic lesions as a major source of these EVs [163].

Novel label-free approaches allowing the quantification and characterization of EVs have demonstrated their potential to improve EV-based diagnosis [164,165,166]. These include atomic force microscopy (AFM) and localized surface plasmon resonance (LSPR). AFM is a surface imaging technique based on a sharp tip mounted on a cantilever that scans samples with nanometer resolution; thus, it allows not only analysis of the structure and morphology of EVs, but also the targeting of surface markers using an antibody-coated AFM tip. LSPR relies on changes in the refractive index in the vicinity of nanoparticles from surface plasmons excited with an incident light beam. The binding of a biomolecular target of interest to a bioreceptor on the nanoparticle surface perturbs the local dielectric environment and leads to a shift of the LSPR peak to a higher wavelength transmission peak that can be monitored with photo-spectrometry. This highly sensitive technique is accurate enough to detect single molecular interactions, including antigen–antibody interactions. Both approaches have allowed successful identification of a high-sensitivity exosomal MCT1 (Monocarboxylate transporter 1) and CD147 (cluster of differentiation 147, basigin) in an experimental mouse model of glioblastoma [167].

### 4.3. EV Nucleic Acid Cargo as Biomarker for the Diagnostic and Prognostic of CRC

Analysis of ncRNA cargo in EVs allows an easy and convenient approach for the diagnosis and follow-up of colorectal cancer (Table 2). This includes the cargo of mRNAs, miRNAs that exhaust selectively target mRNAs and lcnRNAs (including circRNAs) that act by sponging miRNAs or by regulating transcription through epigenetic modulation or interaction with transcription factors [168]. Extracellular vesicle miRome analysis, using the small RNA sequencing of blood samples of multi-stage and longitudinal cohorts, identified EV-miR-320c as a biomarker of metastatic colorectal cancer [103]. High-throughput RNA sequencing of colorectal cancers matched with corresponding control tissue showed the downregulation of circLPAR1, a circRNA generated from circularization of exons 3 and 4 of the lysophosphatidic acid receptor 1 (LPAR1) transcript. Interestingly, the plasma level of exosomal circLPAR1 was significantly decreased in patients with colorectal tumors in a stage dependent manner (polyps vs. adenocarcinomas) and significantly raised after colorectal tumor resection. Mechanistically, exosomal circLPAR1 is up-taken by colorectal cancer cells and binds eIF3h, leading to decreased accumulation of BRD4 (bromodomain containing 4), to inhibition of cell proliferation and to invasiveness. This circRNA might constitute a reliable selective biomarker for the diagnosis of CRC (expression pattern distinct from other types of cancers), for patient follow-up and as a putative therapeutic approach because it reverts some cancer cell phenotypes [125]. One interesting point that is not addressed in the study concerns the source of exosomal circLPAR1 under physiological conditions and the mechanisms related to this downregulation in patients with cancers (local decrease in tumor, in healthy tissue or a systemic decrease). Interestingly, using isogeneic colon cancer cell lines, Dou et al. reported that circRNAs are more abundant in exosomes than in cancer cells themselves, and they also reported that activated KRas downregulated circRNA accumulation [169].

Analysis of circulating DNA in exosomes further allows delineation of some specific cancer cell mutations more efficiently than cell-free DNA. The protection provided by EVs against DNA shearing and degradation sensitizes mutation detection. This is of peculiar importance for precision medicine to delineate patients eligible for such therapy, e.g., wild-type RAS for EGFR inhibitor therapeutic strategies [170]. This is especially valuable for when tissue biopsy in metastatic sites is impossible and for following potential changes in the mutation status of subgroups of cancer cells in the course of treatment.

## 5. Extracellular Vesicles and Colorectal Cancer Cells’ Response to Conventional and Targeted Therapies

The selection of colon cancer sublines resistant to oxaliplatin (L-OHP) has allowed identification of a series of ncRNAs that confer resistance and can alleviate sensitivity in the parental cell lines. These include miR-31-5p, which depletes LATS2 (large tumor suppressor kinase 2), and miR- 46146, which exhausts PDCD10 implicated in apoptosis [171,172]. Based on integrative bioinformatics analysis, the lnCRNAs H19, UCA1 and HOTAIR are also involved in oxaliplatin resistance [173]. MiR-92a-3p and the lncRNA CCAL, carried by exosomes from CAFs, abrogate the CRC cell response to oxaliplatin and to the antimetabolite 5-fluorouracil (5-FU). MiR-92a-3p targets FBXW7 and MOAP1, leading to the activation of the Wnt signaling pathway and the inhibition of mitochondrial apoptosis, respectively, whereas CCAL activates Wnt signaling through interaction with the RNA binding protein HuR and the stabilization of β-catenin transcripts [88,111]. The exosomal circN4BP2L2 released by CAFs promotes the stemness and chemoresistance of Lovo cells to oxaliplatin. This circRNA interacts with and upregulates the translation initiation factor EIF4A3 and stimulates the PI3K/AKT/mTOR pathway [127]. The exosomal miR-210 secreted by adherent colon cancer HCT-8 cells impairs the mesenchymal–epithelial transition of the subpopulations of these cells that underwent EMT and growth in suspension, and it promotes resistance to oxaliplatin combined with 5-FU [99]. The underlying mechanisms have not yet been investigated.

Other indirect mechanisms of resistance to oxaliplatin were attributed to CRC cell-derived exosomal miR-208b through the expansion of immunosuppressive T-Reg cells [60].

The protein cargo contained in EVs also contributes to resistance to oxaliplatin. The heat shock DNAJB8 (DnaJ homolog subfamily B member 8) protein is overexpressed in sublines of the colon cancer SW-480 and SW-620 cells resistant to oxaliplatin and confers resistance to the parental sensitive cells. Mechanistically, DNAJB8 interacts and inhibits the ubiquitination and degradation of P53 and upregulates the drug efflux pump MDR1 (multidrug resistance protein 1). Importantly, DNAJB8 levels in sera from patients with CRC are higher than in sera from healthy individuals, and they decreased after tumor resection. DNAJB8 might constitute a biomarker for the response to oxaliplatin chemotherapy [37]. The exosomal Wnt3a protein from CAFs induces reprograming in vitro and in vivo of CRC cells to a cancer stem cell phenotype, providing resistance to oxaliplatin and 5-FU [46].

Regarding 5-FU, the circular RNA circ_0000338 enables the transfer of the chemoresistance of colorectal cancer cells by quenching miR-217 and miR-485-3p [122]. The targets of these miRNAs that are related to 5-FU sensitivity are not yet characterized. Exosomes from the 5-FU-resistant colon cancer HCT8FU cell line contains a high level of isocitrate dehydrogenase 1 (IDH1), a key enzyme involved in glucose metabolism, and imparts 5-FU resistance to sensitive cells by increasing intracellular levels of NADPH [40]. A similar approach using colon cancer RKO cells identified p-Stat3 as cargo involved in resistance against 5-FU [45]. MiR-21 carried in CAF exosomes was also shown to protect CRC cells against 5-FU. Among the known targets of this miRNA (PDCD4, TPM1 and PTEN), PDCD4 seems to be involved in this protective effect [78].

An indirect mechanism of resistance to SN38, the active metabolite of irinotecan was also evidenced. Active p-ERK and p-AKT proteins in CRC-derived exosomes stimulate hepatic stellate cells to secrete IL6. In turn, IL6 enhances lactate metabolism of hypoxic tumor cells through the STAT3 pathway and upregulation of downstream MCT1 and LDHB, leading to resistance to SN38 [44].

Regarding radiotherapy, the exosomal miR-19b released by colon cancer cells triggers radioresistance and stemness in vitro and in vivo by downregulating FBXW7, a component of the SCF ubiquitin protein ligase complex, and thus, it activates the Wnt/β-catenin signaling pathway [76]. Exosomes derived from CAFs also support CRC resistance to radiotherapy. This process has been attributed to the transfer of miR-93-5p that targets FOXA1 and leads to upregulation of TGFβ [89], whereas miR-590-3p exhausts CLCA4, resulting in the activation of the PI3K/AKT signaling pathways [107].

Extracellular vesicles were also associated with impaired responses to targeted therapies. Circulating lncRNA UCA1-containing exosomes in CRC patients can predict the clinical outcome of the cetuximab anti-EGFR treatment. Furthermore, this lncRNA, which is released by cancer cells, provides resistance to sensitive cells [117]. This process might be related to the sequestration of miR-495, which depletes the receptor tyrosine kinase MET and its ligand, HGF [118]. Similarly, exosomes derived from the cetuximab-resistant RKO cells confer resistance to cetuximab-sensitive Caco-2 cells by downregulating the tumor suppressor PTEN, resulting in the downstream activation of the AKT signaling pathway [174].

## 6. Exosomes for Colorectal Cancer Nanotherapy

Emerging therapeutic strategies using nanomedical approaches aim to enhance drug bioavailability, circumvent the multi-drug resistance of cancer cells and decrease adverse effects and dose-limiting toxicities [21,175]. In this regard, the potential use of engineered exosomes is attracting increasing attention. The intrinsic biocompatibility of exosomes, their stability in blood circulation, their tiny size that enables deep tissue penetration, the stealth and protection they provide to their encapsulated material and their ability to cross plasma membranes make them suitable candidate nanocarriers of therapeutic agents. Alternatively, they could also be used to boost immune response as a cancer vaccine.

Nevertheless, different challenges are to be tackled before exosomes can be used for cancer treatment. These include the source of these exosomes, their production and purification, the loading of bioactive agents, selective cell targeting and long-term storage. High-scale production could be achieved using cell cultures, and alternative sources, including plants, fruits and bovine milk, are also under investigation. The concentration and purification of exosomes can be performed with differential or density gradient centrifugations, nonspecific precipitation using polyethylene glycol, ultrafiltration, chromatography or affinity purification [176,177,178]. These technical approaches suffer distinct drawbacks, e.g., low scale, high time consumption and low purity of exosomes for ultracentrifugation, the latter requiring further purification to obtain clinical-grade exosomes. Extended characterization and quality control are prerequisite for the therapeutic use of manufactured exosomes.

Special attention was devoted to optimizing exosome loading with bioactive agents. These include (i) the isolation of exosome from cell lines treated with a chemotherapeutic agent, (ii) the use of expression vectors allowing overexpression and packaging of nucleic acids, native or chimeric proteins and iii) the loading of purified exosomes using mechanical approaches including calcium phosphate precipitation, electroporation, lipofection, sonication, freeze and thaw cycles and chemical modification [176,178,179,180,181]. Further engineering improvements concern modification of exosome surfaces to favor cell type-selective targeting [179]. The pharmacokinetics, biodistribution and bioavailability of these engineered exosomes will require deeper investigation, and care should also be taken concerning the risk of coisolated endogenous viruses or contamination with pathogens [176,182].

Nevertheless, the efficiency of therapeutic exosomes was successfully assessed in preclinical studies and clinical trials. Concerning colorectal cancers, a series of studies provided the proof of concept for the imaging and delivery of chemotherapeutic agents and/or transfer of miRNAs promoting chemosensitivity.

### 6.1. Preclinical Studies

Several reports have demonstrated the antitumor activity of encapsulated ncRNAs in exosomes. Exosomal transfer of miR-1915-3p impairs the EMT of colon cancer cells and improves their sensitivity to oxaliplatin by suppressing the EMT-promoting oncogenes PFKFB3 and USP2 [183]. Similarly, delivery of miR-204-5p decreases proliferation, induces apoptosis and enhances the response to 5-FU of LoVo and HCT116 colon cancer cells in vitro and in vivo by targeting RAB22A and Bcl2 [98]. The circRNA F-box and WD repeat domain containing 7 (circ-FBXW7) is decreased in CRC and resistant to oxaliplatin treatment. The delivery of circ-FBXW7 to SW-480 and HCT-116 cell derivatives resistant to oxaliplatin restores cancer cells’ sensitivity both in vitro and in experimental mouse models through sequestration of miR-18b-5p [124]. MiR-34a-loaded tumor exosomes originating from mouse colon CT-26 cancer cells reduce the growth of CT-26 tumors in Balb/c mice not only by acting on cancer cells themselves, but also by inducing T cell polarization toward CD8+ T subsets among tumor-infiltrating lymphocytes [85,86]. 

The selective conveying of doxorubicin to colon cancer cells approached by encapsulating doxorubicin in tumor-derived exosomes from the human colorectal carcinoma LIM1215 cell line covered with antibodies directed against A-33, a cell surface glycoprotein overexpressed on colorectal cancer cells, produced promising results on tumor growth in nude mice [184]. Milk extracellular vesicles loaded with oxaliplatin and conjugated with GE11 peptide to target cells expressing EGFR were efficiently delivered, and they triggered apoptosis in vitro of the human cecum cancer SNU-C5 cells and markedly affected the growth of these cancer cells as xenografted in nude mice [185]. The engineering of tumor exosomes derived from the human colon cancer HCT-116 cell line and loaded with 99mTc and Cy7 probes was demonstrated in an experimental mouse model. Their higher uptake by cancer cells compared to exosomes produced by adipose stem cells gained interest in such a strategy for SPECT/NIRF tumor imaging [186]. Macrophage M1-derived extracellular vesicles loaded with the photosensitizer zinc phthalocyanine enhance the efficiency of photodynamic therapy compared to M2, melanoma or milk -derived EVs through the modulation of immune response in MC38 tumor-bearing mice [187].

Exosomes enable the combined delivery of chemotherapeutic agents and ncRNAs. The lncRNA PGM5-AS1 is downregulated in colorectal cancer compared to control mucosa, and this downregulation is associated with oxaliplatin resistance. PGM5-AS1 acts as an hs-miR-423-5p sponge, leading to the upregulation of the nucleoside diphosphate kinase NME1. Exosomes derived from HEK-293 cells overexpressing PGM5-AS1 and loaded with oxaliplatin reverse colon cancer cells’ resistance to oxaliplatin in vitro and in vivo after subcutaneous xenografts in nude mice [110]. 

Kwon et al. developed exosome-based hybrid nanostructures by decorating exosome surfaces derived from human colon cancer HT-29 cells with both folic acid (FA) as a tumor-targeting ligand, taking advantage of FA receptor overexpression on cancer cells, and magnetic nanoparticles coupled to EpCam for a hyperthermia therapy using alternating magnetic fields [188]. These engineered exosomes were further loaded with doxorubicin. This combined chemotherapy/hyperthermia therapy efficiently impaired the tumor growth of HT-29 cells xenografted in nude mice without apparent toxicity on mouse organs [188].

Combined strategies of diagnosis by imaging and therapy, termed theranostic, are also under investigation. Exosomes produced by the human colorectal cancer HCT116 cells loaded with doxorubicin as therapeutic agents and 68Ga-L-NETA-DBCO allowing PET imaging were successfully delivered in orthotopic xenografts of colon cancer cells in mice [189].

### 6.2. Clinical Trials

Phase I clinical trials using engineered exosomes are under way (Table 3). The high affinity of hydrophobic drugs with exosomes derived from many fruits allows circumventing the major obstacles of their use in clinic, which are due to their poor stability, solubility and bioavailability. In this context, a phase I clinical trial of curcumin conjugated with plant exosomes administrated as a dietary supplement to patients with colorectal cancer prior to tumor resection is ongoing. Curcumin, the main component of curry, exhibits antitumor activity on colon cancer cells in vitro, in experimental mouse models and in patients with colorectal cancers [190]. The deliverables of this trial concern the impact of exosomally delivered curcumin on immune modulation, cellular metabolism and the phospholipid profiles of normal and malignant colon cells.

Another ongoing phase I trial being performed on patients with metastatic colorectal cancers involves exosomes loaded with a synthetic lipid-tagged Stat6 antisense oligonucleotide: exoASO-STAT6 (CDK-004) (Table 3). Accordingly, a preclinical study performed on syngeneic mouse models of colorectal cancer demonstrated that this monotherapy decreases tumor growth by more than 90% and results in 60% complete remission. These exosomes, which are produced by human kidney embryonic HEK293 cells overexpressing prostaglandin F2 receptor negative regulator (PTGFRN), proved to have a tropism for tumor-associated macrophages. The delivery of Stat6 antisense oligonucleotides to these M2 immune-suppressive macrophages triggers their reprogramming towards the proinflammatory M1 phenotype, resulting in remodeling of the tumor microenvironment and generation of a CD8 T cell–mediated adaptive immune response [191].

**Table 3:** Clinical trial concerning investigations on extracellular vesicles for the diagnosis, prognosis or treatment of colorectal cancer.

Type, Origin of Exosomes	Official Title of the Study	Biological, Clinical Parameters	Type of Cancer	Type	Clinical Trials.gov Identifier
Exosomes from peripheral venous blood drawn immediately prior to surgery	A Study of Imaging, Blood, and Tissue Samples to Guide Treatment of Colon Cancer and Related Liver Tumors	Diagnosis of colon cancer, prognosis of spreading to other organs	Colon	Observational	NCT03432806
Exosomes from blood sample	Identification of New Diagnostic Protein Markers for Colorectal Cancer	Number, size and protein composition of blood exosomes; diagnosis of colorectal cancer	Colorectal	Observational	NCT04394572
Exosomes from serum samples of patients before, during and after chemoradiation therapy	Exosomes in Rectal Cancer	Exosomal biomarkers assessment; exosomal expression; functional evaluation of exosomes in malignant colonic organoids and mouse models of colorectal cancer	Rectal	Observational	NCT03874559
Exosome RNA from peripheral blood samples before chemoradiotherapy	Study on Predictive Biomarkers of Neoadjuvant Chemoradiotherapy for Rectal Cancer	Biomarkers for response and toxicity to neoadjuvant therapy; treatment optimization	Rectal	Observational	NCT04227886
Protein content and tumor DNA in extracellular vesicles from blood of patients before during and after chemoradiation therapy	A Prospective Feasibility Study Evaluating Extracellular Vesicles Obtained by Liquid Biopsy for Neoadjuvant Treatment Response Assessment in Rectal Cancer	Biomarkers of the response of rectum cancer to neoadjuvant treatment	Rectal	Observational	NCT04852653
Exosomes from stored blood samples of patients diagnosed with colorectal cancer between 2008 and 2012	Contents of Circulating Extracellular Vesicles: Biomarkers in Colorectal Cancer Patients (ExoColon)	Prognosis value of size, number and content (protein, lipid, RNA…) of circulating exosomes	Colorectal	Observational	NCT04523389
Exosomes from serum samples of patients following psychological intervention	Impact of Group Psychological Interventions on Extracellular Vesicles in People Who Had Cancer (MindGAP-P)	Impact of psychological intervention on the blood concentration of extracellular vesicles and patient outcome	Breast, colorectal cancer	Observational	NCT04298398
Levels of the cytokines IL-6, IL-8, IL-10 and IL-12 in microparticles and serum	The Relationship Between Relaxation or Wheat Germ Juice to the Immune Indices and Quality of Life (QoL) in Colorectal Cancer Patients on Adjuvant Chemotherapy	Impact of physical and psychological well-being on proinflammatory cytokine levels in patients with colorectal cancer	Colorectal cancer stage II or III treated with adjuvant chemotherapy (capecitabine or FU-5 treatment, conjoined with oxaliplatin or capecitabine alone following curative surgery)	Observational	NCT01991080
Blood microparticles	Microparticles in Peritoneal Carcinomatosis of Colorectal Origin	Characterization of microparticulate signature from the blood of patients with peritoneal carcinomatosis of colorectal origin; comparison with colorectal cancer without peritoneal carcinomatosis	Colorectal cancer patient with or without peritoneal carcinosis	Clinical trial, early diagnosis of peritoneal carcinomatosis of colorectal cancer	NCT03969784
Changes of PD-L1 expression on exosomes in peripheral blood after treatment of patients with toripalimab	Phase II Study of Toripalimab Plus Stereotactic Body Radiotherapy in Colorectal Cancer Patients with Oligometastasis	Response to immunotherapy (immune checkpoint inhibition)	Metastatic colorectal cancer	Clinical trial, phase 2	NCT03927898
Exosomes from serum samples of patients before, during and after radiotherapy	Early Biomarkers of Tumor Response in High Dose Hypofractionated Radiotherapy Word Package 3: Immune Response	Biomarker of immune response	Liver metastasis from colorectal cancer, hepatocarcinoma	Clinical trial	NCT02439008
Exosomes from serum samples of patients during treatment	Tyrosine Kinase Inhibitor (TKI) + Anti-PD-1 Antibody in TKI-responded Microsatellite Stability/Proficient Mismatch Repair (MSS/pMMR) Metastatic Colorectal Adenocarcinoma	Safety of tyrosine kinase inhibitor in combination with anti-PD-1 antibody in TKI-responded MSS metastatic colorectal adenocarcinoma	Metastatic colorectal cancer	Clinical trial, phase 2	NCT04483219
Measurement of tissue factor-bearing microparticles (tumor origin)	Enoxaparin Thromboprophylaxis in Cancer Patients with Elevated Tissue Factor Bearing Microparticles (MicroTEC)	Evaluation of the efficiency of enoxaparin in preventing blood clots in the veins in participants who have cancer and also have high levels of tissue factor bearing microparticles in their blood	Metastatic colorectal, pancreas, nonsmall cell lung, ovary, gastric	Clinical trial, observation, prevention	NCT00908960
Blood microparticles	Cancer Associated Thrombosis and Isoquercetin (CATIQ)	Efficiency of isoquercetin to prevent venous thromboembolic events in cancer patients	Colorectal (stage IV), pancreas, nonsmall cell lung cancer	Clinical trial, phase II/III, prevention	NCT02195232
Curcumin conjugated with plant exosomes	Study investigating the ability of plant exosomes to deliver curcumin to normal and colon cancer tissue	Impact on immune modulation, cellular metabolism, and phospholipid profile; normal and malignant colon cells from who are undergoing surgery for newly diagnosed colon cancer	Colon	Clinical trial, phase 1	NCT01294072
Cell-derived exosomes loaded with a synthetic lipid-tagged STAT6 antisense oligonucleotide	A Study of exoASO-STAT6 (CDK-004) in Patients with Advanced Hepatocellular Carcinoma (HCC) and Patients with Liver Metastases from Primary Gastric Cancer and Colorectal Cancer (CRC)	Reprogramming of immune suppressive M2 macrophage to proinflammatory M1 phenotype with potential for meaningful antitumor activity	Liver metastases from gastric and colorectal cancer, hepatocarcinoma	Clinical trial, phase 1	NCT05375604

## 7. Conclusions

The crosstalk between cancer cells, stromal cells, immune cells and distant target tissues as well as the molecular actor diversity illustrate the complexity of the spatiotemporal events leading to cancer progression and the metastatic cascade, and they highlight the difficult challenge of their analysis. Extracellular vesicles open up great prospects not only in the holistic characterization of these intercellular communications but also in their interest for the early and noninvasive detection of colorectal cancer, for better follow-up and improved patient care and for their potential use as therapeutic vectors. Databases compiling the cargo identified in EVs along with their cellular origins (e.g., EVAtlas, http://bioinfo.life.hust.edu.cn/EVAtlas/#/ (accessed on 16 December 2022) for ncRNA profiles in EVs from different tissues and biological fluids; Vesiclepedia, http://www.microvesicles.org/ (accessed on 16 December 2022) for proteins, RNA and lipids, last updated 2018) constitute powerful tools to delineate the specificity of novel potential biomarkers. Nevertheless, although many studies report the identification of promising biomarkers for the diagnosis and prognosis of colorectal cancers, none of them have been so far validated for translation to the clinic. This gap might be connected to different factors, including the redundancy of some players (such as ncRNAs), the diversity of experimental models, the higher level of complexity of the EV repertoire from tumor tissue compared to cultured cells, the cohort sizes, the disease stage, tumor heterogeneity and the EV isolation, purification and storage methodologies discussed above, but also, more importantly, the technical approaches that can be implemented in routine clinical practice. The development of integrated microfluidic technologies integrating biosensors and allowing high-throughput and high-sensitivity detection of specific biomarkers, including protein and nucleic acids from human blood without purification steps, should enable efficient and noninvasive diagnoses and follow-ups for patients with CRCs at an affordable cost [192,193,194,195,196,197]. As far as EVs are concerned for CRC treatment, besides their potential use as theranostic vector, it is also conceivable to develop strategies to counteract their oncogenic activity by inhibiting their release, by targeting the machineries involved in cargo sorting or by acting on EV cargo themselves.

Further studies are required for a better understanding of the mechanisms underlying the selective cargo packaging, how EVs orchestrate intercellular communications, and how these go awry in cancer. This opens up the promise of not only earlier and better diagnoses of CRCs but also some avenues for novel therapeutic strategies.
