# Correction: Kotelevets, L.; Chastre, E. Extracellular Vesicles in Colorectal Cancer: From Tumor Growth and Metastasis to Biomarkers and Nanomedications. *Cancers* 2023, *15*, 1107

**DOI:** 10.3390/cancers17050806

**Published:** 2025-02-26

**Authors:** Larissa Kotelevets, Eric Chastre

**Affiliations:** Sorbonne Université, INSERM, UMR_S938, Centre de Recherche Saint-Antoine (CRSA), 75012 Paris, France

**References**

After the publication of our Review article [1], two of the referenced manuscripts were withdrawn. A further evaluation of all the references, performed in November 2024, enabled us to identify additional papers of questionable validity.

To enhance the quality of our manuscript, we have decided to remove the suspicious references from this corrected version. The reference citations have been updated to reflect the revised reference list.

This correction was approved by the Academic Editor, and the authors state that their scientific conclusions are unaffected. The original publication has also been updated.
